# ERRα and HIF-1α Cooperate to Enhance Breast Cancer Aggressiveness and Chemoresistance Under Hypoxic Conditions

**DOI:** 10.3390/cancers17142382

**Published:** 2025-07-18

**Authors:** Dimas Carolina Belisario, Anna Sapino, Ilaria Roato, Amalia Bosia, Sophie Doublier, Serena Marchiò

**Affiliations:** 1 Department of Oncology, University of Torino, 10060 Candiolo, Italy; dimascarolina.belisario@unito.it (D.C.B.); amalia.bosia@unito.it (A.B.); 2 Candiolo Cancer Institute, Fondazione del Piemonte per l’Oncologia—Istituto di Ricovero e Cura a Carattere Scientifico (FPO-IRCCS), 10060 Candiolo, Italy; anna.sapino@unito.it; 3 Department of Medical Sciences, University of Turin, 10100 Turin, Italy; 4 Department of Surgical Sciences, University of Turin, 10100 Turin, Italy; ilaria.roato@unito.it; 5 Centre de Recherche des Cordeliers, Institut National de la Santé et de la Recherche Médicale (INSERM), Sorbonne Université, 75006 Paris, France; sophie.doublier@sorbonne-universite.fr

**Keywords:** breast cancer, hypoxia, multidrug resistance

## Abstract

**Background/Objectives**: HIF-1α and ERRα are both implicated in breast cancer progression, yet their functional interplay remains poorly understood. This study investigates their molecular crosstalk in the context of hypoxia-induced drug resistance. **Methods**: MCF-7 (estrogen receptor, ER-positive) spheroids and CoCl_2_-treated SK-BR-3 (ER-negative) cells were used to model tumor hypoxia. Protein expression, coimmunoprecipitation, chromatin immunoprecipitation (ChIP), pharmacological inhibition, and siRNA-mediated gene silencing were employed to assess physical and functional interactions. Immunohistochemistry (IHC) on a tissue microarray (TMA) of 168 invasive breast carcinomas was performed to evaluate clinical relevance. **Results**: ERRα levels remained unchanged under hypoxia, while its coactivator, Peroxisome Proliferator-Activated Receptor Gamma Coactivator-1 α (PGC-1α), was upregulated. ERRα physically interacted with HIF-1α and was required for HIF-1 transcriptional activity under hypoxic conditions. ChIP assays showed that ERRα-driven overexpression of Permeability glycoprotein 1 (P-gp) and Vascular Endothelial Growth Factor (VEGF) was mediated by HIF-1α binding to the *MDR1* and *VEGF* promoters. Inhibition or silencing of ERRα reversed P-gp overexpression and restored intracellular doxorubicin. TMA analysis confirmed the clinical correlation between ERRα, HIF-1α, and P-gp expression, highlighting the role of ERRα in hypoxia-induced drug resistance. ERRα expression was independent of ER status, suggesting an estrogen-independent function. **Conclusions**: This study identifies a novel physical and functional interaction between ERRα and HIF-1α that promotes chemoresistance in hypoxic breast tumors. Targeting ERRα may represent a promising therapeutic strategy to overcome drug resistance in aggressive, ER-independent breast cancer subtypes.

## Simple Summary

Hypoxia within breast tumors promotes resistance to chemotherapy, complicating treatment strategies. This study characterizes a functional interaction between Estrogen-Related Receptor α (ERRα) and Hypoxia-Induced Factor α (HIF-1α) that enhances drug resistance and tumor aggressiveness under hypoxia. Additionally, it shows that targeting ERRα may overcome resistance and improve treatment outcomes, independent of hormone receptor status.

## 1. Introduction

Breast cancer remains a leading cause of cancer-related death among women worldwide [1,2]. Despite significant advancements in early detection and treatment [3], resistance to therapy, particularly neoadjuvant chemotherapy, continues to pose a major challenge in clinical management. A critical factor contributing to this resistance is the development of an MDR phenotype, which reduces the efficacy of chemotherapeutic agents and leads to poor clinical outcomes [4,5]. It has long been recognized that hypoxic conditions within the tumor microenvironment play a key role in the onset of MDR, promoting cancer cell stemness [6] and inducing angiogenesis [7]. The transcription factor HIF-1α is central to these processes [8], as adaptation to hypoxia is largely mediated through a HIF-1α–dependent transcriptional program [9].

More recently, the orphan nuclear receptor ERRα has attracted attention for its role in breast cancer [10,11,12,13,14]. Unlike classical ERs, ERRα does not bind to estrogens but shares target genes and coregulators with them [15]. In preclinical models of human breast cancer, ERRα has been shown to induce and maintain stemness, leading to increased invasiveness and drug resistance [16,17,18]. Accordingly, elevated levels of ERRα in patients are associated with more aggressive tumor phenotypes, higher recurrence rates, and reduced overall survival [19,20,21,22,23,24,25].

Studies on human umbilical vascular endothelial cells [26] and breast cancer cell lines [27] have established a link between ERRα and the cellular response to hypoxic stress. These studies revealed that ERRα stimulates the expression and secretion of the angiogenesis inducer VEGF by directly binding to and activating the *VEGF* gene promoter, independent of HIF-1α. However, another study revealed that ERRα physically interacts with and activates HIF-1α in a genetically controlled cell model of prostate cancer [28]. This finding is consistent with that of *Ao* et al., who first reported that members of the ERR family physically interact with HIF-1α and stimulate HIF-induced transcription in different cancer cell lines [29]. The functional connections between ERRα and HIF-1α have also been conceptualized in a recent review focused on metabolic pathways in endometrial cancer [30]. These discrepancies suggest that the interactions between ERRα and HIF-1α are complex and not yet fully understood.

In this study, we investigated the molecular and functional interactions between HIF-1α and ERRα in breast cancer models and clinical samples to identify potential therapeutic strategies for enhancing treatment efficacy and overcoming drug resistance.

## 2. Materials and Methods

### 2.1. Cell Culture and Treatments

The human breast cancer cell lines MCF-7 (RRID: CVCL_0031) and SK-BR-3 (RRID: CVCL_0033) were obtained from ATCC (Manassas, VA, USA) and maintained in phenol red-free DMEM/Ham’s F12 medium (Gibco, Thermo Fisher Scientific, Waltham, MA, USA) supplemented with 10% FBS, 100 U/mL each of penicillin and streptomycin, and 1% L-glutamine (Sigma Aldrich, St. Louis, MO, USA). The cell lines were authenticated by Short Tandem Repeats (STRs) profiling upon receipt and were reauthenticated every 6 months. Mycoplasma contamination was routinely monitored using the MycoAlert™ Mycoplasma Detection Kit (Lonza, Basel, Switzerland; RRID: SCR_008925) and confirmed to be negative before the experiments. For normoxic culture, the cells were grown as monolayers in a humidified incubator at 37 °C with 5% CO_2_ and 20% O_2_. To mimic hypoxic conditions, SK-BR-3 cells were treated with 375 µM CoCl_2_, and spheroids of MCF-7 cells were generated as previously described [31]. Briefly, 5 × 10^6^ cells were seeded onto 100 mm dishes in DMEM/Ham’s F12 medium containing 1% FBS and 20 μg/mL human neutrophil elastase (VWR, Milan, Italy) and maintained in a humidified incubator at 37 °C with 5% CO_2_ and 20% O_2_ for 48 h. Treatments were administered as follows: doxorubicin (Sigma‒Aldrich, D2975000) 3 µM for 18 h, YC-1 (HIF-1α inhibitor, Sigma‒Aldrich, Y102) 2.5 µM, XCT790 (inverse agonist of ERRα, Sigma‒Aldrich, #X4753) 10 µM, and DES (steroidal inhibitor of ERRα, Sigma‒Aldrich, 614289) 25 µM for 24 h.

### 2.2. Western Blot

Protein extracts were prepared using a Nuclear Extraction Kit (Active Motif, Vinci-Biochem, Florence, Italy), and 120 µg of nuclear protein was resolved by 8–15% SDS-PAGE, followed by transfer onto PVDF membranes (Immobilon-P, Millipore, Sigma‒Aldrich). The membranes were blocked by incubation with 5% nonfat dry milk and 0.1% Tween-20 in TBS. They were then incubated for 1 h with the following primary antibodies: mouse monoclonal anti-human HIF-1α, 1:1000 (54/HIF-1α, BD Biosciences, Franklin Lakes, NJ, USA; RRID: AB_398272); rabbit polyclonal anti-ERRα, 1:1000 (07-662, Millipore, Burlington, MA, USA; RRID: AB_310305); mouse monoclonal anti-PCNA, 1:2000 (PC10, Santa Cruz Biotechnology, Dallas, TX, USA; RRID: AB_628110); and mouse monoclonal anti-PGC-1α, 1:500 (1F3.9, Calbiochem, Sigma‒Aldrich; RRID: AB_2239901), followed by incubation with HRP-conjugated anti-rabbit (RRID: AB_11125142) or anti-mouse (RRID: AB_11125547) antibodies (Bio-Rad, Hercules, CA, USA), both 1:20,000. Proteins were detected using enhanced chemiluminescence (Perkin Elmer, Waltham, MA, USA). Band intensity was quantified using ImageJ software version 1.54p [32] and normalized to the PCNA signal.

### 2.3. Coimmunoprecipitation

The cells were washed with cold PBS, followed by lysis in 50 mM Tris-HCl (pH 7.4), 150 mM NaCl, 1% Triton X-100, 50 µg/mL pepstatin, 50 µg/mL leupeptin, 10 µg/mL aprotinin, 1 mM PMSF, 100 µM ZnCl_2_, and 1 mM Na_3_VO_4_ for 30 min. Total proteins (500 µg) were added to 1 mL of detergent-free lysis buffer (50 mM Tris-HCl, pH 7.4, 150 mM NaCl, 50 µg/mL pepstatin, 50 µg/mL leupeptin, 10 µg/mL aprotinin, 1 mM PMSF, 100 µM ZnCl_2_, and 1 mM Na_3_VO_4_) and immunoprecipitated overnight with 10 µg of rabbit polyclonal anti-ERRα antibody (RRID: AB_310305) and 15 µL of preequilibrated protein-G Sepharose (Amersham Pharmacia, Piscataway, NJ, USA). After three washes in ice-cold detergent-free lysis buffer; two washes in 25 mM Tris, 0.5 M LiCl, and 1 mM Na_3_VO_4_; and three washes in 25 mM Tris pH 8.0, 150 mM NaCl, 1 mM EDTA, and 1 mM Na_3_VO_4_, the proteins were separated by 10% SDS‒PAGE and transferred to PVDF membranes.

### 2.4. HIF-1 Activation Assay

HIF-1 activation was assessed by ELISA (TransAM Transcription Factor Activation Assay, Active Motif, Vinci-Biochem, Vinci, Italy) following the manufacturer’s protocol. Briefly, 10 µg of nuclear protein was added to each well of a 96-well plate precoated with a double-stranded oligonucleotide containing the HRE from the *EPO* gene (5′-TACGTGCT-3′). The plate was incubated with an anti-HIF-1α antibody (RRID: AB_397491) followed by an HRP-conjugated anti-mouse antibody (RRID: AB_11125547) diluted in Antibody Binding Buffer. Binding was visualized by incubation with the Developing Solution until a blue color appeared. The absorbance was measured using a Biotek EL340 microplate reader (Agilent Technologies, Santa Clara, CA, USA) at 450 nm, with a reference wavelength of 655 nm, and expressed as mU OD/μg nuclear proteins.

### 2.5. RNA Interference

Transfection of siRNA oligonucleotides was performed in DMEM/Ham’s F12 supplemented with 10% FBS using the jetPRIME transfection reagent (Polyplus, Euroclone, Pero, Italy). The cells were seeded at a density of 3 × 10^5^ cells/well in 6-well plates and treated with 110 pmol of ERRα siRNA (sc-44706) or control siRNA-A (Santa Cruz Biotechnology). At 24 h posttransfection, MCF-7 spheroids and CoCl_2_-treated SK-BR-3 cells were prepared as described above. ERRα silencing was confirmed by semiquantitative PCR.

### 2.6. Immunofluorescence

The cells were fixed in 3.5% paraformaldehyde containing 2% sucrose for 15 min at room temperature and then washed with PBS. Nonpermeabilized cells were blocked with 3% BSA for 1 h at 4 °C and stained overnight at 4 °C with a PE-conjugated mouse monoclonal anti-P-gp antibody (UIC-2, Millipore; RRID: AB_1234567). DAPI (Sigma‒Aldrich) was added for 5 min at room temperature. A 100 µL suspension of cells or spheroids was placed onto glass slides using a cytospin, and the cytocentrifuged cells were dried, mounted with Vectashield mounting medium (Vector Laboratories, Burlingame, CA, USA), and examined using a Zeiss SM 5 Pascal Model confocal microscope (Carl Zeiss International, Oberkochen, Germany). Controls included incubation with the mouse IgG2a isotype control.

### 2.7. Reverse Transcription and Semiquantitative PCR

Total RNA was extracted via TRIzol reagent (Thermo Fisher) according to the manufacturer’s instructions. To ensure removal of genomic DNA contamination, 1 µg of total RNA was subjected to a genomic DNA elimination step followed by reverse transcription using the QuantiTect Reverse Transcription Kit (Qiagen, Germantown, MD, USA), which includes an integrated gDNA wipeout buffer and reverse transcriptase mix. Semiquantitative PCR was carried out to assess the expression of selected target genes. Primer pairs were designed to span exon–exon junctions where possible or flank intronic sequences, to distinguish cDNA products from potential contaminating genomic DNA. PCR reactions were conducted in a final volume of 25 µL containing 1 µL of cDNA, 1× reaction buffer, 0.2 mM dNTPs, 0.4 µM of each primer, and 1.25 U of Taq DNA polymerase (Thermo Fisher). Cycling conditions were optimized for each gene as follows:

(1) Human *MDR1* gene:
  Forward: 5′-GACTGAGCCTGGAGGTGAAG-3′;
  Reverse: 5′-CCACCAGAGAGCTGAGTTCC-3′ (60 °C, 40 cycles).
(2) Human *ESRRA* gene:
  Forward: 5′-TGCCAATTCAGACTCTGTGC-3′;
  Reverse: 5′-CCTCGAGCATCTCCAAGAAC-3′ (60 °C, 31 cycles).
(3) Human *B2M* gene:
  Forward: 5′-AGCAAGGACTGGTCTTTCTATCTC-3′;
  Reverse: 5′-ATGTCTCGATCCCACTTAACTATCTT-3′ (56 °C, 30 cycles).

The derived products were separated on a 1.5% agarose gel containing ethidium bromide and visualized under UV light. Band intensities were quantified using ImageJ software, and expression levels of target genes were normalized to *B2M*. Negative controls (no reverse transcriptase) were included in all experiments to confirm absence of genomic DNA contamination. All PCRs were performed in at least two biological replicates and validated for reproducibility.

### 2.8. ChIP Assay

The cells were incubated with 1% formaldehyde for 10 min at 37 °C to cross-link the proteins to the DNA. The cross-linking reaction was quenched by the addition of a one-tenth volume of 1.25 mM glycine. The cells were washed twice in ice-cold PBS, resuspended in radioimmunoprecipitation assay buffer (150 mM NaCl, 1% NP40, 0.5% deoxycholate, 0.1% SDS, 5 mM EDTA, 50 mM Tris-HCl, pH 8.0) containing 1 mM PMSF, 1 μg/mL aprotinin, and 1 μg/mL pepstatin A, and kept on ice for 30 min. Next, the cell lysates were sonicated on ice with a UP200S ultrasonic sonicator (3 × 40 s, amplitude 40%; Hielscher Ultrasonics GmbH, Teltow, Germany) until the cross-linked chromatin was sheared to yield DNA fragments between 200 and 1000 bp. Sonicates were incubated with salmon sperm DNA/protein agarose-50%. Immunoprecipitation was performed overnight at 4 °C with 5 μg of mouse monoclonal anti-HIF-1α (28b, Santa Cruz Biotechnology; RRID: AB_2129647) or 1.6 μL of rabbit polyclonal anti-HIF-1α–ChIP grade (ab2185, Abcam, Cambridge, UK). The immunoprecipitates were supplemented with 5 M NaCl and heated overnight at 65 °C to reverse protein‒DNA cross-links. The immune complexes were further treated with DNase- and RNase-free proteinase K, and the DNA was purified via phenol/chloroform extraction and ethanol precipitation. PCR was performed using the following primers to amplify:

(1) The HRE within the promoter region of the human *MDR1* gene:
  Forward: 5′-GGAGCAGTCATCTGTGGTGA-3′
  Reverse: 5′-CTCGAATGAGCTCAGGCTTC-3′
(2) An *MDR1* promoter region that does not contain an HRE:
  Forward: 5′-GAAGGTCTTCCCAGTAACCTACC-3′
  Reverse: 5′-GCCAGAGTTGAGAAGTTTAGCC-3′
(3) The HRE within the promoter region of the human *VEGF* gene:
  Forward: 5′-GCCTCTGTCTGCCCAGCTGC-3′
  Reverse: 5′-GTGGAGCTGAGAACGGGAAGC-3′
(4) A *VEGF* promoter region that does not contain an HRE:
  Forward: 5′-TGGAGAGAAGGAGGAAGGGG-3′
  Reverse: 5′-CCAGTGGAAGGGGAAGGAA-3′

The PCR products were separated on a 4% agarose gel and quantified with ImageJ.

### 2.9. Measurement of Doxorubicin Accumulation

Intracellular doxorubicin levels were measured via a fluorometric assay. The cells were incubated with 3 µM doxorubicin for 18 h, washed with PBS, and resuspended in 1 mL of a 1:1 mixture of ethanol and 0.3 NHCl. The samples were sonicated on ice using a UP200S ultrasonicator (1 × 10 s, amplitude 40%). The doxorubicin content was measured using a Perkin-Elmer LS-5 fluorimeter at 475 nm for excitation and 553 nm for emission. The fluorescence readings were converted to nanograms of doxorubicin per milligram of cellular protein via a calibration curve.

### 2.10. TMA Staining

The BR2085d TMA was purchased from TissueArray.com. This TMA contained 144 cases of invasive carcinoma of no special type, 24 invasive lobular carcinomas, 1 breast tissue, and 19 adjacent normal tissues. The pathology grade, TNM, clinical stage, and pathological markers (ER, PR, HER2, and Ki67) were obtained from the manufacturer. The TMA slides were baked at 65 °C (dry heat) for 1 h, followed by deparaffinization in two changes of 100% xylene and rehydration in a graded ethanol series to distilled water. To block endogenous peroxidase activity, the slides were incubated in a 3% hydrogen peroxide solution (Merck KGaA, Darmstadt, Germany) for 20 min. Antigen retrieval was performed by heating the slides in citrate buffer (pH 6.0; Histo-Line Laboratories, Milan, Italy) in a microwave for 2 × 7 min. Nonspecific protein binding was blocked with normal goat serum blocking solution (Vector Laboratories, Milan, Italy) for 1 h. Slides were incubated overnight at 4 °C with the following primary antibodies (all from Thermo Fisher): ESRRA polyclonal antibody, 1:500 (PA5-28749; RRID: AB_2546225); HIF1A monoclonal antibody (mgc3) 1:20 (MA1-516; RRID: AB_2150546); PGC1α alpha polyclonal antibody, 1:100 (bs-1832R; RRID: AB_10856845); and P glycoprotein polyclonal antibody, 1:300 (22336-1-AP; RRID: AB_2833023). Following incubation with either anti-mouse-HRP (RRID: AB_2617137) or anti-rabbit-HRP (RRID: AB_2617138) (both Agilent DAKO, Santa Clara, CA, USA), detection was performed with 3, 3′-diaminobenzidine (DAB) for 10 min. Slides were counterstained with hematoxylin, rinsed in distilled water, dehydrated through a graded ethanol series, cleared with two changes of 100% xylene, and mounted in Bio-Mount mounting medium (Bio Optica, Milan, Italy). Images were acquired with a MorphoLens 6 slide scanner (Morphle Labs, New York, NY, USA) and processed with QuPath version 0.5.1 [33]. DAB signals were quantified using ImageJ and verified in a blinded manner by an operator to correct for tissue deterioration or staining anomalies where necessary. IHC scores were assigned based on the intensity range for each marker on a scale of 0 (lowest value) to 4 (highest value).

### 2.11. Statistical Analysis

Statistical analysis of the in vitro experiments was conducted using GraphPad Prism (version 10.0.0; GraphPad Software, Boston, MA, USA). The results are presented as the means ± standard deviations from at least two independent experiments, each performed in triplicate. Statistical significance was assessed using paired *t* tests, with a threshold of *p* < 0.05. The TMA staining results were analyzed using Python (version 3.11) to perform statistical evaluations and generate correlation matrices. To aid in exploratory data interpretation and results summarization, we used ChatGPT 4.0 as a language model–based assistant for drafting preliminary descriptions and guiding code development, under human supervision. Spearman’s rank correlation coefficients were calculated to assess the associations between ERRα, PGC-1α, HIF-1α, P-gp, and clinicopathological variables. These coefficients measure the strength and direction of the relationships between two variables and range from −1 to +1. Positive values indicate that as one variable increases, the other tends to increase, whereas negative values suggest an inverse relationship. For regression analysis, OLS multiple linear regression models were constructed to evaluate predictive relationships. Regression analysis determines how well one variable predicts another variable. Each marker was treated as a dependent variable in separate models, and the remaining markers served as independent predictors. Model fit was assessed using β and R^2^ values. The β coefficient represents the strength and direction of the relationship between the predictors and the dependent variable (positive β: the predictor increases the dependent variable; negative β: the predictor decreases it). The R^2^ value indicates the proportion of variance in the dependent variable explained by the model, ranging from zero to one, with higher values suggesting a better fit. Statistical significance was determined using *p* values (*p* < 0.05). Heatmaps were generated in Python using Matplotlib (version 3.7.1).

## 3. Results

### 3.1. ERRα and HIF-1α Interact Under Hypoxic Conditions in Breast Cancer Cell Lines

**Figure 1:**
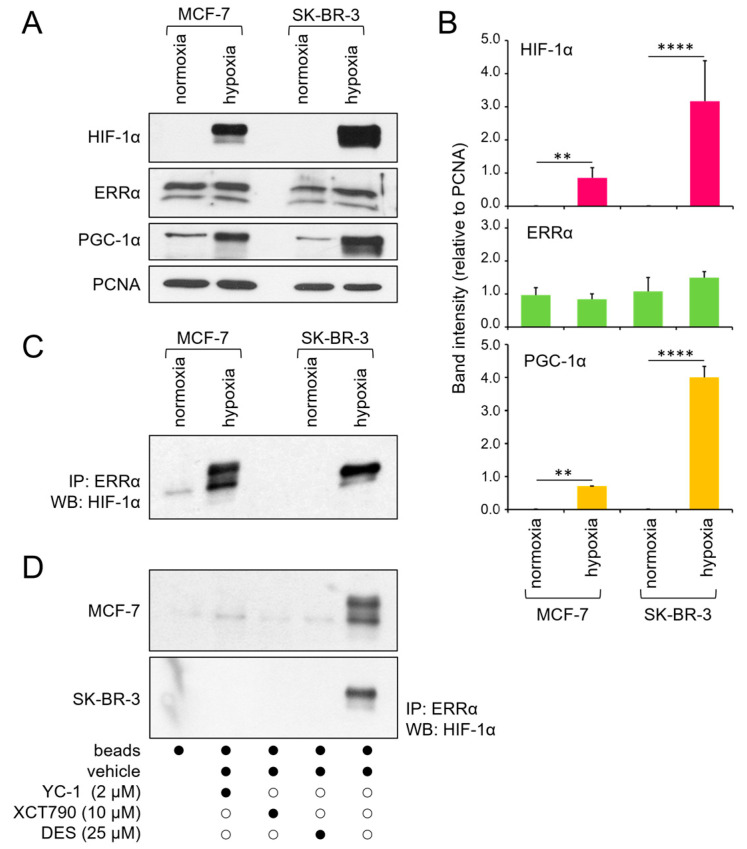
Expression and physical interaction of ERRα and HIF-1α. (**A**) Western blot analysis of HIF-1α, ERRα, and PGC-1α protein levels in the nuclear extracts of MCF-7 and SK-BR-3 cells under normoxic or hypoxic conditions. PCNA was used as a nuclear protein loading control. (**B**) Quantification of band intensity from 3 independent experiments, normalized to that of PCNA and expressed as the mean ± SD. Statistical analysis by paired *t* test: ****, *p* < 0.0001; **, *p* < 0.01 (hypoxia vs. normoxia). (**C**) Coimmunoprecipitation of ERRα and HIF-1α under the same culture conditions. (**D**) Coimmunoprecipitation of ERRα and HIF-1α under hypoxia following a 24 h incubation with the indicated inhibitors (black dots represent the presence, and white dots the absence, of the treatment listed on the left). Normoxia, monolayer cell culture in 20% O_2_; hypoxia, spheroid (MCF-7) or CoCl_2_ treatment (SK-BR-3); IP, immunoprecipitation; WB, Western blot. The uncropped blots are shown in File S1.

To explore the molecular mechanisms underlying ERRα-mediated breast cancer aggressiveness, we initially examined the expression of ERRα and its coactivator PGC-1α at the protein level in two human breast cancer cell lines with distinct ER statuses, MCF-7 (ER-positive) and SK-BR-3 (ER-negative), under different oxygenation conditions. Monolayers cultured in 20% O_2_ were used as reference (normoxic) conditions for both cell lines. To reproduce the hypoxia typically observed in the inner regions of solid tumors, MCF-7 cells were cultured as multicellular spheroids. Since SK-BR-3 cells do not form spheroids [34], hypoxia was simulated in this cell line by CoCl_2_ treatment [35]. Western blot analyses of nuclear protein extracts revealed no difference in ERRα levels across various culture conditions. In contrast, PGC-1α was markedly upregulated in both cell lines under hypoxic conditions, consistent with the expected increase in HIF-1α levels (Figure 1A,B).

Previous studies have indicated that HIF-1α physically interacts with ERRα in prostate cancer cells [28]. Considering the potential significance of this interaction in malignant progression, we investigated whether it is also present in breast cancer cells. Coimmunoprecipitation assays confirmed that HIF-1α and ERRα proteins specifically bind to each other under hypoxic conditions in both the MCF-7 and SK-BR-3 cell lines (Figure 1C). Additionally, we observed that this interaction occurs exclusively when both proteins are active, as they are disrupted in the presence of specific functional inhibitors, namely, YC-1 for HIF-1α and DES and XCT790 for ERRα (Figure 1D).

### 3.2. The Interaction Between ERRα and HIF-1α Is Necessary for HIF-1α Activation

**Figure 2:**
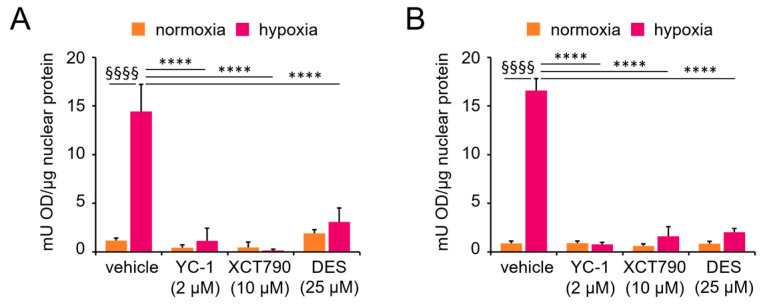
Functional interaction between ERRα and HIF-1α. HIF-1α transcriptional activity was evaluated by ELISA in nuclear extracts from (**A**) MCF-7 and (**B**) SK-BR-3 cells cultured under normoxic (monolayer) or hypoxic conditions (spheroid or CoCl_2_ treatment) for 24 h, with or without ERRα or HIF-1α inhibitors. Values are the means ± SDs from two independent experiments, each in triplicate. Statistical analysis by paired *t* test: §§§§, *p* < 0.0001 (hypoxia vs. normoxia in the absence of treatments); ****, *p* < 0.0001 (vehicle vs. treatments under hypoxia).

Next, we evaluated HIF-1 activity using an ELISA-based assay that quantifies the ability of the HIF-1α subunit to bind a double-stranded oligonucleotide containing the HRE from the *EPO* gene. As expected, HIF-1α recruitment to its response element was strongly increased in both cell lines under hypoxic conditions, and this recruitment was reversed by the specific inhibitor YC-1. Notably, treatment with the ERRα inhibitors XCT790 and DES also markedly reduced the levels of DNA-bound HIF-1α. In contrast, no significant changes were observed under normoxic conditions, where basal levels of DNA-bound HIF-1α remained unaffected by the inhibitors (Figure 2). These results are consistent with those of the coimmunoprecipitation experiments, confirming that ERRα is required for HIF-1-dependent transcriptional functions.

### 3.3. ERRα Is Necessary for HIF-1-Mediated Overexpression of the P-gp Protein

**Figure 3:**
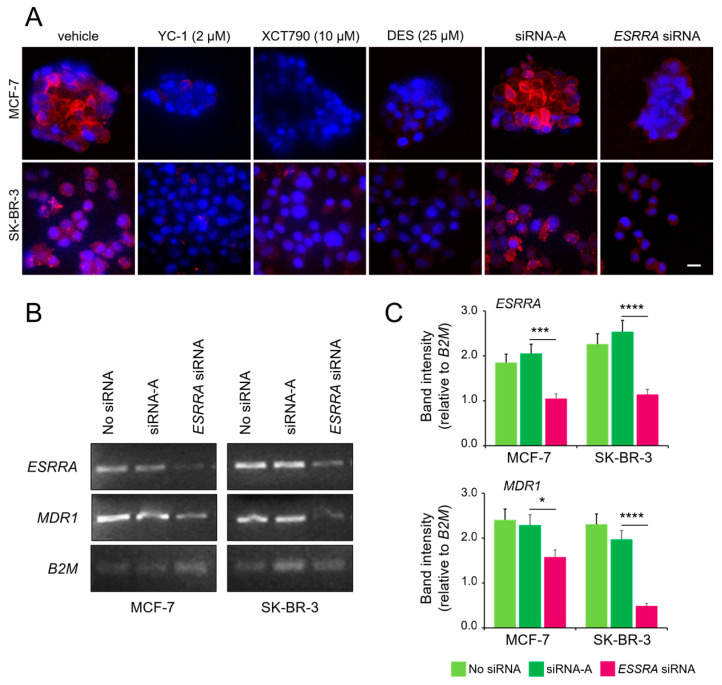
ERRα is necessary for HIF-1α-mediated overexpression of the P-gp protein. (**A**) Immunofluorescence confocal microscopy showing P-gp expression in MCF-7 (spheroid) and SK-BR-3 (CoCl_2_-treated) cells, following 24 h treatment with YC-1, XCT790, DES, or after transfection with an *ESSRA*-targeted siRNA. Cells were stained with PE-conjugated anti-P-gp (red) and counterstained with DAPI (blue). Images are representative of three independent replicates. Some MCF-7 spheroid-derived cells may appear without visible nuclei due to DAPI diffusion limits or sectioning artifacts. Scale bar, 20 μm. (**B**) Semi-quantitative PCR analysis of *ESRRA* (ERRα), *MDR1* (P-gp), and *B2M* (housekeeping gene used for normalization of gel loading) from hypoxic cells with or without *ESRRA* siRNA knockdown and (**C**) quantification of band intensities normalized to those of *B2M*. The values represent the means ± SDs of two independent experiments. Statistical analysis by paired *t* test: ****, *p* < 0.0001; ***, *p* < 0.001; *, *p* < 0.05 (ESRRA-targeted siRNA vs. control siRNA). Vehicle only (jetPRIME transfection reagent) and an untargeted siRNA (siRNA-A) were used as negative controls in these experiments. The uncropped blots are shown in File S1.

We previously demonstrated that HIF-1 activation increases the expression of P-gp, leading to drug resistance in MCF-7 cells under hypoxic conditions [31]. To investigate the potential role of ERRα in this process, we used two approaches: pharmacological inhibition and posttranscriptional silencing of *ESRRA* (the gene encoding ERRα protein). Confocal fluorescence microscopy confirmed the presence of P-gp in the plasma membrane of both cell lines under hypoxic conditions. Treatment with YC-1 (HIF-1α inhibitor, used as a control), XCT790, or DES (ERRα inhibitors), as well as *ESRRA* silencing, prevented P-gp expression and membrane localization (Figure 3A). To corroborate these results, semiquantitative PCR analysis of total RNA from MCF-7 and SK-BR-3 cells under hypoxic conditions revealed significant downregulation of the P-gp protein-coding gene *MDR1* when *ESRRA* gene expression was downregulated by the targeted siRNA (Figure 3B,C). Together, these findings suggest the presence of a functionally active ERRα for the activation of the drug resistance phenotype under hypoxic conditions.

### 3.4. ERRα Is Necessary for HIF-1α Binding to the MDR1 and VEGF Gene Promoters

**Figure 4:**
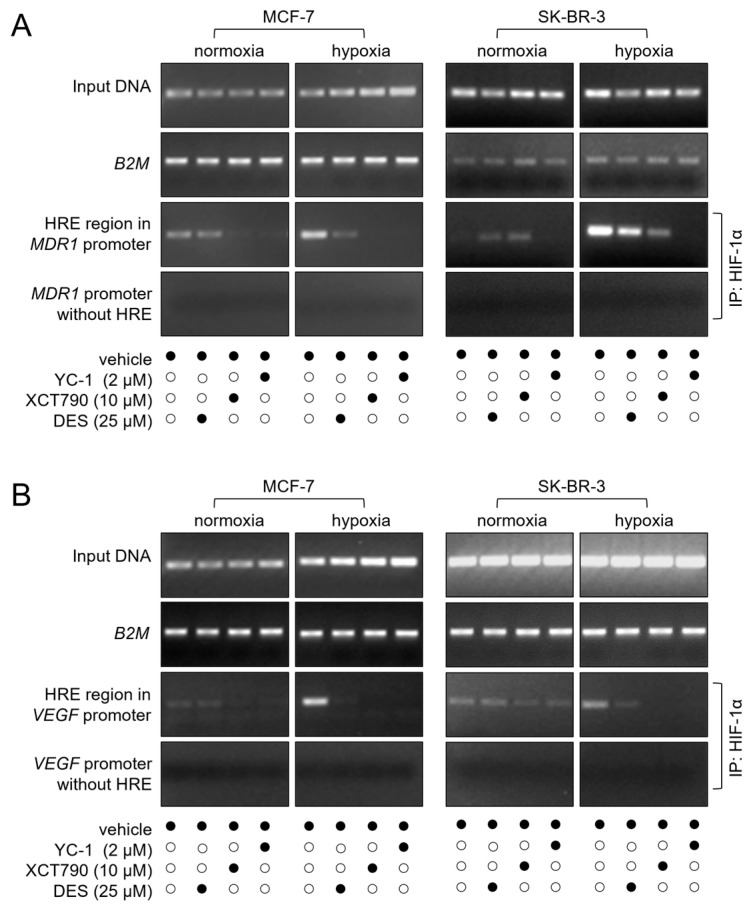
ERRα is required for HIF-1α binding to the *MDR1* and *VEGF* promoters. (**A**) HIF-1α binding to the *MDR1* promoter was assessed by ChIP and visualized as a PCR-amplified fragment from either a complete promoter region or an HRE-deleted (negative control) region. (**B**) HIF-1α binding to the *VEGF* gene promoter was evaluated using the same experimental approach. In both (**A**) and (**B**), the input DNA and *B2M* gene amplification (used for normalization of gel loading) are also shown. The cells were treated with the indicated concentrations of the inhibitors for 24 h (black dots represent the presence, and white dots the absence, of the treatment listed on the left). Normoxia, monolayer cell culture in 20% O_2_; hypoxia, spheroid (MCF-7), or CoCl_2_ treatment (SK-BR-3). IP, immunoprecipitation. The figures are representative of two independent experiments. The uncropped blots are shown in File S1.

We further investigated the mechanism of the ERRα/HIF-1α-driven overexpression of the P-gp protein at the transcriptional level. Additionally, we explored whether the observed upregulation of the *VEGF* gene induced by ERRα in endothelial cells [26] and breast cancer cells [27] could also be mediated by HIF-1α. For this purpose, we performed a ChIP to test the ability of HIF-1α to bind to the HRE within the *MDR1* and *VEGF* gene promoters in the MCF-7 and SK-BR-3 cell lines under hypoxic conditions. As expected, in both cases, a strong band was detected by immunoprecipitation with an anti-HIF-1α antibody, followed by PCR amplification of either the *MDR1* or *VEGF* gene promoter. These results suggest that HIF-1α may contribute to the transcriptional regulation of drug resistance and angiogenesis-associated genes under hypoxic conditions. As anticipated, the presence of the HIF-1α inhibitor YC-1 inhibited binding to either promoter. Remarkably, treatment with the ERRα inhibitors DES and XCT790 also impaired HIF-1α binding, underscoring the functional connection between ERRα and HIF-1α in *MDR1* and *VEGF* transcriptional activation (Figure 4).

### 3.5. ERRα Inhibition Restores Intracellular Doxorubicin Retention Under Hypoxia

**Figure 5:**
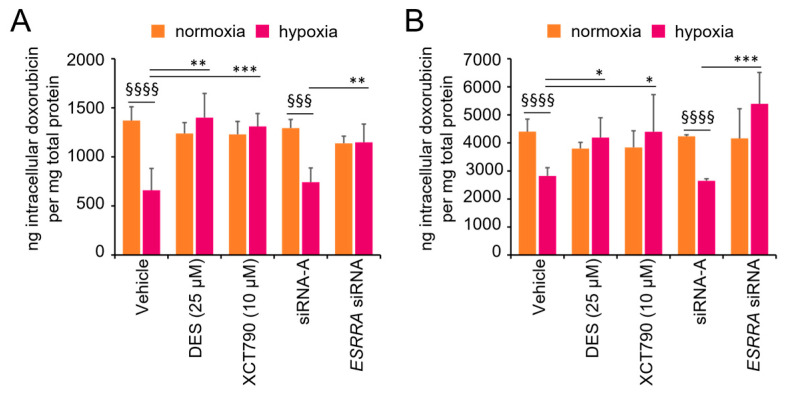
ERRα inhibition restores intracellular doxorubicin retention under hypoxia. Intracellular doxorubicin was quantified by a fluorometric assay in (**A**) MCF-7 or (**B**) SK-BR-3 cells under normoxic or hypoxic conditions with ERRα inhibitors (DES, XCT790) or an *ESRRA*-targeting siRNA. Vehicle only (jetPRIME transfection reagent) and an untargeted siRNA (siRNA-A) were used as negative controls. Fluorescence values were normalized to protein content. The values are the means ± SDs of two independent experiments performed in triplicate. Statistical analysis by paired *t* test: §§§§, *p* < 0.0001; §§§, *p* < 0.001 (hypoxia vs. normoxia in both control experimental points); ***, *p* < 0.001; **, *p* < 0.01; *, *p* < 0.05 (vehicle vs. treatments under hypoxic conditions). No difference was observed between vehicle and the untargeted siRNA experimental points. Normoxia, monolayer cell culture in 20% O_2_; hypoxia, spheroid (MCF-7) or CoCl_2_ treatment (SK-BR-3).

P-gp protein, which is overexpressed under hypoxic conditions, induces drug resistance by promoting drug efflux, leading to decreased intracellular accumulation of doxorubicin [31]. Since we observed that ERRα inhibitors reversed P-gp overexpression, we investigated whether they could also restore intracellular doxorubicin retention as an indirect indicator of regained drug sensitivity. For this purpose, we treated MCF-7 and SK-BR-3 cells with 3 µM doxorubicin for 18 h in combination with the ERRα inhibitors DES and XCT790 or after transfection with the *ESRRA*-targeting siRNA. Measurement of intracellular drug accumulation revealed that both functional inhibition and posttranscriptional downregulation of ERRα protein levels restored the cellular retention of doxorubicin under hypoxic conditions (Figure 5). As expected, the amount of intracellular doxorubicin was not affected by these treatments under normoxic conditions, where P-gp expression is known to be present at very low levels [31].

### 3.6. ERRα Expression Is Clinically Correlated with PGC-1α, HIF-1α, and P-gp Expression

**Figure 6:**
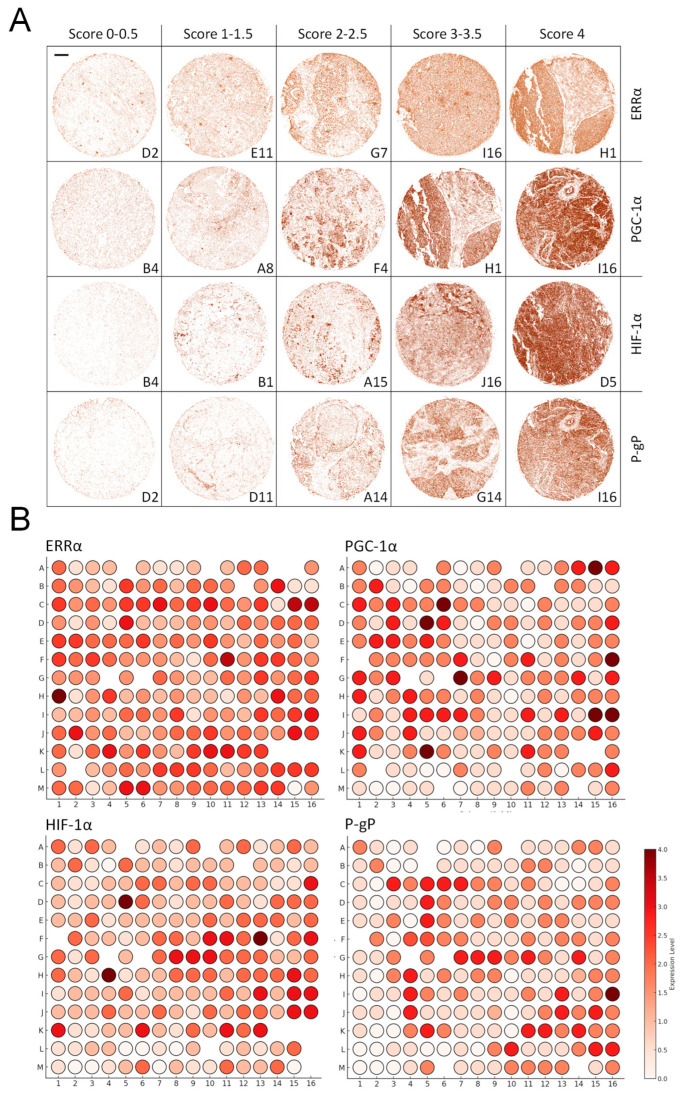
Expression of ERRα, PGC-1α, HIF-1α, and P-gp in a TMA of invasive breast cancer. (**A**) Immunohistochemical staining for each marker in representative TMA cores. Signal intensity (DAB chromogen) was scored on a 0–4 scale. Images show examples of low, moderate, and high expression levels. A scale bar corresponding to 100 μm has been inserted in the upper left corner of the first image. (**B**) Heatmaps display staining scores for each marker across 168 invasive breast carcinoma samples and 19 adjacent tissues. Missing data points correspond to damaged or lost tissue sections. Quantification was performed with ImageJ, and spot validation was conducted by a blinded pathologist.

To validate the in vitro findings in a clinical setting, we analyzed the protein expression of ERRα, PGC-1α, HIF-1α, and P-gp in a TMA containing 144 invasive carcinoma cases and 24 invasive lobular carcinoma cases (Figure 6, Supplementary Table S1).

The staining intensity was scored on a scale from 0 to 4, and Spearman’s correlation analysis was performed to assess the relationships between these markers and clinicopathological features (Table 1). Clinically, ERRα expression was not significantly associated with ER or PR status, further confirming that its role in breast cancer progression and hypoxia adaptation is independent of estrogen receptor signaling. HIF-1α expression was significantly greater in high-grade and low-PR tumors (*p* < 0.05), reflecting its well-established role in aggressive breast cancer phenotypes [9,36].

Correlation analysis of the four IHC markers (Table 2) revealed robust interrelationships between ERRα, PGC-1α, HIF-1α, and P-gp, supporting our in vitro results on their roles in hypoxia-driven transcription and drug resistance [28,29].

Specifically, (1) a strong positive correlation between ERRα and PGC-1α (r = 0.50, *p* < 0.001) indicates that ERRα expression is closely linked to PGC-1α levels, reinforcing their role as coregulators; (2) a moderate positive correlation between ERRα and HIF-1α (r = 0.38, *p* < 0.01) indicates that ERRα may contribute to the activation of HIF-1α under hypoxic conditions, potentially influencing pathological outcomes; (3) a moderate positive correlation between ERRα and P-gp (r = 0.40, *p* < 0.001) supports the hypothesis that ERRα regulates drug resistance by influencing P-gp expression; and (4) a strong positive correlation between HIF-1α and P-gp (r = 0.49, *p* < 0.001) confirms the assumption that HIF-1α is a key driver of P-gp overexpression under hypoxia. These findings corroborate the notion that ERRα contributes to drug resistance both through HIF-1α-mediated transcription of the *MDR1* gene and independently.

To further explore the interdependencies between the IHC markers, multiple linear regression analysis was performed with each marker as a dependent variable and the remaining three as independent predictors (Table 3).

The analysis revealed that (1) ERRα levels were significantly predicted by PGC-1α (β = 0.30, *p* < 0.001) and HIF-1α (β = 0.17, *p* < 0.03), with R^2^ = 0.29, suggesting that these markers are closely linked to ERRα activity under hypoxic conditions; (2) PGC-1α expression was strongly predicted by ERRα (β = 0.38, *p* < 0.001) and P-gp (β = 0.46, *p* < 0.001), with R^2^ = 0.44, supporting its role as a coactivator in hypoxia-driven transcription; (3) HIF-1α levels were significantly predicted by ERRα (β = 0.22, *p* < 0.03) and P-gp (β = 0.35, *p* < 0.001), with R^2^ = 0.32, confirming the contribution of ERRα to HIF-1α activation; and (4) P-gp expression was best predicted by a combination of ERRα, PGC-1α, and HIF-1α (R^2^ = 0.41, *p* < 0.001), underscoring their collective role in driving drug resistance. Collectively, these findings offer clinical evidence that ERRα collaborates with HIF-1α to promote hypoxia-induced P-gp expression, contributing to breast cancer chemoresistance. This interplay highlights the intricate regulatory network governing hypoxia-driven cancer progression and suggests that targeting ERRα could be a promising strategy to overcome multidrug resistance in hypoxic tumors.

## 4. Discussion

This study highlights the role of ERRα in promoting breast cancer aggressiveness under hypoxic conditions via its interaction with HIF-1α. We demonstrated that ERRα not only binds to HIF-1α but is also functionally required for its activation and subsequent transcription of key genes involved in drug resistance and angiogenesis, specifically *MDR1* (which encodes P-gp) and *VEGF*. Importantly, the inhibition of ERRα significantly reduced HIF-1α activity and reversed doxorubicin efflux typical of the MDR phenotype, providing strong support for the targeting of ERRα in hypoxia-driven, chemoresistant breast tumors.

Our molecular interaction studies align with the findings of Ao et al. [29], who demonstrated that members of the ERR family physically interact with HIF-1α and stimulate HIF-induced transcription in cancer cell lines. While previous reports [6,9,26,27,29] have separately linked ERRα and HIF-1α to hypoxic responses, our work uniquely demonstrates their interconnected activity in a 3D spheroid model of breast cancer. Furthermore, we provide a mechanistic perspective on the interaction between ERRα and HIF-1α, showing that, under hypoxic conditions, the expression of PGC-1α, a known ERRα coactivator, is significantly increased despite stable ERRα levels. This suggests a compensatory mechanism through which cancer cells may enhance ERRα activity under oxygen deprivation, as PGC-1α is a potent coactivator that amplifies ERRα-driven transcriptional responses [37,38]. Accordingly, increased expression of PGC-1α has also been observed in an ovarian cancer model following an energy crisis induced by genetic or pharmacological impairment of Complex I in the oxidative phosphorylation [39]. This hypothesis aligns with known feedback mechanisms [40] and calls for further investigation into coactivator-mediated regulation of ERRα function and its impact on breast cancer aggressiveness.

One of the most significant findings of our study is the role of ERRα in the hypoxia-driven overexpression of P-gp, which facilitates drug resistance by promoting drug efflux. While previous studies have established that HIF-1α enhances P-gp expression in cancer cells [6,9], our results reinforce these findings by demonstrating that ERRα is essential for this process. We showed that ERRα inhibition, either through pharmacological means or via siRNA, reduces P-gp expression and restores doxorubicin retention into both ER-positive and ER-negative cells. While increased intracellular doxorubicin accumulation is indicative of reduced drug efflux, we did not directly assess cytotoxicity or treatment sensitivity (e.g., changes in IC_50_ values), and functional resensitization to doxorubicin will be confirmed in future studies.

In addition to its role in drug resistance, the ERRα–HIF-1α interaction is critical for *VEGF* gene upregulation, supporting the contribution of ERRα to hypoxia-driven angiogenesis. The involvement of ERRα in *VEGF* regulation has been documented in endothelial [26] and breast cancer cells [27,29]; however, our study is the first to show that inhibiting ERRα disrupts HIF-1α binding to the *VEGF* promoter in breast cancer cells. These findings further implicate ERRα as a key player in the angiogenic response of hypoxic breast tumors. Given that tumor vascularization supports both nutrient supply and drug resistance, targeting ERRα may provide dual benefits: inhibiting both blood vessel-sustained tumor growth and chemoresistance.

Our TMA analysis bridges the gap between mechanistic in vitro findings and clinical relevance by demonstrating that ERRα expression positively correlates with PGC-1α and HIF-1α in breast cancer tissues. These findings reinforce the hypothesis that ERRα acts as a coregulator of HIF-1α-driven transcriptional programs in hypoxic tumors. The observed correlation between ERRα and P-gp further supports the role of ERRα in mediating drug resistance [11,12,30,31], which is a major challenge in the treatment of aggressive breast cancer. The role of ERRα in chemoresistance is likely multifactorial and may extend beyond the regulation of P-gp. While our study focused on the ERRα–HIF-1α axis and its downstream impact on *MDR1* expression, ERRα has also been implicated in broader metabolic rewiring. Prior studies show that ERRα overexpression confers resistance to lapatinib, tamoxifen, and doxorubicin [10,12,14] by modulating glycolysis, fatty acid oxidation, and mitochondrial biogenesis. Thus, ERRα may contribute to drug resistance even in tumors where P-gp is not a primary mediator. Further investigations across a spectrum of chemoresistant models—including those with P-gp-independent mechanisms—will be valuable to delineate the full extent of ERRα contribution.

Importantly, we found no correlation between ERRα expression and ER status, suggesting that ERRα-driven hypoxic responses are not limited to hormone receptor-positive tumors. Our experiments did not directly assess TNBC models; however, the ER-independent activity of ERRα and its correlation with HIF-1α observed in clinical samples support its potential relevance in this aggressive subtype, which is often highly hypoxic [41], exhibits stem-like properties [42], and is notoriously chemoresistant [43].

Our study did not include in vivo models; however, we sought to bridge this gap using two complementary approaches: 3D spheroid cultures and a large breast cancer TMA. The use of spheroids better replicates the hypoxic tumor microenvironment compared to 2D cultures, including heterogeneous hypoxia and possible necrotic cores, and enables the study of hypoxia-driven transcriptional responses in a physiologically relevant context. Furthermore, the strong positive correlations observed among ERRα, HIF-1α, PGC-1α, and P-gp in the TMA samples support the translational relevance of our in vitro findings. Although direct assessment of hypoxia markers such as carbonic anhydrase IX or pimonidazole was not feasible in this dataset, the expression of HIF-1α—a canonical marker of hypoxic signaling—serves as a surrogate indicator of hypoxia-associated transcriptional activity. These converging lines of evidence support our proposed model of ERRα-mediated hypoxia adaptation, although future in vivo studies are warranted for full validation.

The inhibition of ERRα impacts multiple downstream pathways, including those regulated by HIF-1α, thereby increasing the potential value of ERRα-directed therapies in treating aggressive breast cancer. Despite this promising foundation, and although several ERRα inhibitors—such as those used in this study—have shown preclinical efficacy, no ERRα-targeted therapies are currently under evaluation in clinical trials. Ongoing efforts by academic and pharmaceutical groups (e.g., Lead Pharma) have produced potent, selective ERRα antagonists, but these compounds have yet to reach investigational new drug (IND)-enabling studies. The slow translation of ERRα-targeted therapies to the clinic stems from several challenges: (i) The ligand-binding domain is partially occluded, complicating small molecule design [44]; (ii) functional redundancy with ERRβ and ERRγ raises concerns about selectivity; and (iii) because ERRα may exert distinct functions depending on the tissue context—such as metabolic regulation in muscle versus transcriptional coactivation in tumors—predicting its therapeutic relevance and selecting responsive patient subgroups remains a challenge. Nevertheless, structure-guided drug design and emerging insights into ERRα synthetic lethality with oncogenic pathways are reinvigorating drug development [45,46,47]. Importantly, mouse models show that genetic deletion or pharmacological inhibition of ERRα impairs mitochondrial metabolism without inducing systemic toxicity [48], suggesting a favorable therapeutic window. However, formal toxicology studies and human data are still lacking.

In addition to supporting the development of new therapeutic strategies, our findings, along with prior preclinical studies, suggest several promising contexts also for the exploitation of EERα for diagnostic and prognostic purposes. In TNBC, ERRα overexpression supports proliferation and metabolic reprogramming, positioning it as a candidate marker for therapies targeting tumor energetics [23]. In post-neoadjuvant residual disease, increased ERRα expression correlates with chemoresistance and may help identify patients at higher recurrence risk who could benefit from targeted intervention [49]. In endocrine-resistant ER-positive tumors, ERRα may substitute for ER signaling, promoting survival via alternative transcriptional programs. Thus, ERRα expression may serve as a surrogate biomarker of endocrine resistance, informing treatment strategies [50]. These scenarios underscore the need for prospective studies to validate ERRα as a clinically actionable biomarker.

## 5. Limitations

This study has a few limitations. First, although the hypoxia models employed—CoCl_2_ treatment in monolayers and spheroid culture—recapitulate key features of the tumor microenvironment, they do not fully reflect the spatial and cellular complexity of in vivo hypoxia. In addition, although both models induced robust HIF-1α stabilization and consistent downstream responses, their differences in dimensionality and oxygen sensing may introduce distinct molecular adaptations that do not entirely overlap with those occurring in vivo. Second, while we observed consistent results using both pharmacological inhibitors and siRNA-mediated knockdown of ERRα, the possibility of off-target effects from the small-molecule inhibitors cannot be entirely excluded. In particular, we cannot rule out that the observed loss of interaction between ERRα and HIF-1α—especially following YC-1 treatment—may partially reflect reduced HIF-1α protein levels in addition to the impairment of a functional complex. Third, although the TMA provided valuable insights into marker co-expression in human breast cancers, it lacked direct hypoxia markers and clinical outcome data, which limits its utility for prognostic validation. Moreover, although a strong positive correlation was observed between ERRα and PGC-1α, this does not constitute definitive evidence of functional coactivation in the context of HIF-1α-driven transcription in patients. Our regression analysis also indicates that PGC-1α may exert independent effects on P-gp expression, apart from its interaction with ERRα. Finally, while our findings are supported by both 3D cell models and clinical tissue analysis, we acknowledge the absence of in vivo data confirming the spatial co-localization of ERRα and hypoxia-related markers. Future validation in orthotopic or patient-derived xenograft models will be necessary to confirm the relevance of this regulatory axis under physiological conditions.

## 6. Conclusions

In summary, our study establishes ERRα as a critical co-regulator of HIF-1α–driven transcription under hypoxic conditions in breast cancer, promoting both *VEGF* gene induction and chemoresistance through P-gp upregulation. Through comprehensive in vitro analyses and validation in a large panel of patient-derived tissue samples, we demonstrate that ERRα physically interacts with HIF-1α and is indispensable for its activation and DNA-binding capacity at key promoter regions. The inhibition or silencing of ERRα not only disrupts this interaction but also restores doxorubicin retention in breast cancer cells, highlighting the therapeutic potential of targeting ERRα to counteract MDR. Importantly, the effects of ERRα are independent of ER status, suggesting its relevance across diverse breast cancer subtypes, including aggressive, ER-negative tumors such as TNBC. Given the clinical significance of these findings, future research should focus on developing selective pharmacological inhibitors that effectively block ERRα function in hypoxic tumors. These inhibitors could provide promising approaches to combat chemoresistance and angiogenesis in aggressive breast cancer subtypes. Additionally, clinical trials will be necessary to determine whether ERRα can serve as a predictive biomarker of treatment response, especially in drug-resistant, hypoxia-driven tumors.

## Figures and Tables

**Table 1:** Spearman correlation coefficients (r) and *p* values for the associations between IHC markers and clinical variables. Statistically significant values are highlighted in bold.

		Grade	ER Score	ER %	PR Score	PR %	HER2	Ki67 %
**(r)**	**ERRα**	0.041653	−0.12126	−0.10895	−0.15221	−0.15907	−0.054690	−0.138250
	**PGC-1α**	0.042560	−0.11219	−0.10497	−0.12734	−0.12082	−0.051130	0.019983
	**HIF1α**	**0.181403**	−0.12506	−0.15319	**−0.19829**	**−0.18007**	0.028343	0.026184
	**P-gp**	0.052622	−0.02616	−0.02247	−0.04643	−0.04213	0.023183	0.062225
***p*** **value**	**ERRα**	0.645997	0.179733	0.228398	0.091483	0.077627	0.54630	0.125716
	**PGC-1α**	0.638830	0.214768	0.245942	0.158734	0.181326	0.572743	0.825645
	**HIF1α**	**0.043767**	0.166382	0.089388	**0.027268**	**0.045365**	0.754679	0.772835
	**P-gp**	0.561618	0.773019	0.804377	0.608611	0.642197	0.798283	0.492363

**Table 2:** Correlation coefficients (r) and *p* values between the IHC markers. All values are statistically significant.

		ERRα	PGC-1α	HIF1α	P-gp
**(r)**	**ERRα**	1	0.504028	0.379303	0.403852
	**PGC-1α**	0.504028	1	0.40845	0.546257
	**HIF1α**	0.379303	0.40845	1	0.491339
	**P-gp**	0.403852	0.546257	0.491339	1
***p*** **value**	**ERRα**		2.41 × 10^−9^	1.40 × 10^−5^	3.30 × 10^−6^
	**PGC-1α**	2.41 × 10^−9^		2.49 × 10^−6^	5.30 × 10^−11^
	**HIF1α**	1.40 × 10^−5^	2.49 × 10^−6^		6.86 × 10^−9^
	**P-gp**	3.30 × 10^−6^	5.30 × 10^−11^	6.86 × 10^−9^	

**Table 3:** Regression analysis of potential dependencies. Statistically significant values are highlighted in bold.

Dependent Variable	Predictor	β Coefficient	*p* Value	R^2^
ERRα	PGC-1α	**0.296600358**	**0.000161052**	**0.293983474**
ERRα	HIF1α	**0.173441476**	**0.029272525**	**0.293983474**
ERRα	P-gp	0.058089575	0.526283255	0.293983474
PGC-1α	ERRα	**0.378690390**	**0.000161052**	**0.439191513**
PGC-1α	HIF1α	0.145059442	0.10823915	0.439191513
PGC-1α	P-gp	**0.462682551**	**3.04 × 10^−6^**	**0.439191513**
HIF1α	ERRα	**0.224748095**	**0.029272525**	**0.31746520**
HIF1α	PGC-1α	0.147223266	0.10823915	0.31746520
HIF1α	P-gp	**0.354564763**	**0.000498647**	**0.31746520**
P-gp	ERRα	0.057750214	0.526283255	0.407189879

## Data Availability

The data generated in this study are available upon request from the corresponding author.

## Supplementary Materials

The following supporting information can be downloaded at: https://www.mdpi.com/article/10.3390/cancers17142382/s1, Table S1: Description of TMA-included samples and IHC scores for ERRα, PGC-1, HIF-1α, and P-gP; File S1: Full pictures of the Western blots.

## Abbreviations

The following abbreviations are used in this manuscript:

*B2M*	Human β-2 microglobulin
DAB	3,3′diaminobenzidine
DAPI	4′,6-diamidino-2-phenylindole dihydrochloride
DES	Diethyl-stilbestrol
*EPO*	Erythropoietin gene
ER	Estrogen receptor
ERRα	Estrogen-related receptor alpha protein
*ESRRA*	Estrogen-related receptor alpha gene
IHC	Immunohistochemistry
IND	Investigational new drug
HIF-1α	Hypoxia-inducible factor 1-alpha
HRE	Hypoxia‒response element
*MDR1*	Multidrug resistance 1 gene
OLS	Ordinary least squares
PCNA	Proliferating cell nuclear antigen
PE	Phycoerythrin
PGC-1α	Peroxisome proliferator-activated receptor-γ coactivator 1 alpha
P-gp	Permeability glycoprotein 1
STR	Short tandem repeat
TMA	Tissue microarray
TNBC	Triple-negative breast cancer
VEGF	Vascular endothelial growth factor
