# Supplementary material for: ERRα and HIF-1α Cooperate to Enhance Breast Cancer Aggressiveness and Chemoresistance Under Hypoxic Conditions
Source: Cancers (Basel). 2025 Jul 18;17(14):2382. doi: 10.3390/cancers17142382 (PMC12293820; doi:10.3390/cancers17142382)
Supplement: Supplementary file 1 [file cancers-17-02382-s001.zip › cancers-3728766-File S1.pdf]

ERRα and HIF-1α Cooperate to Enhance Breast Cancer Aggressiveness and Chemoresistance Under Hypoxic Conditions

Blots and Images

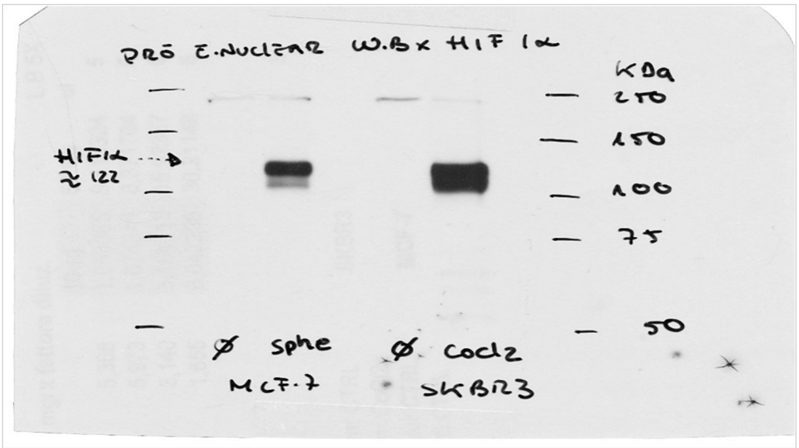

Full uncropped blot from **Fig. 1A**, HIF-1α.

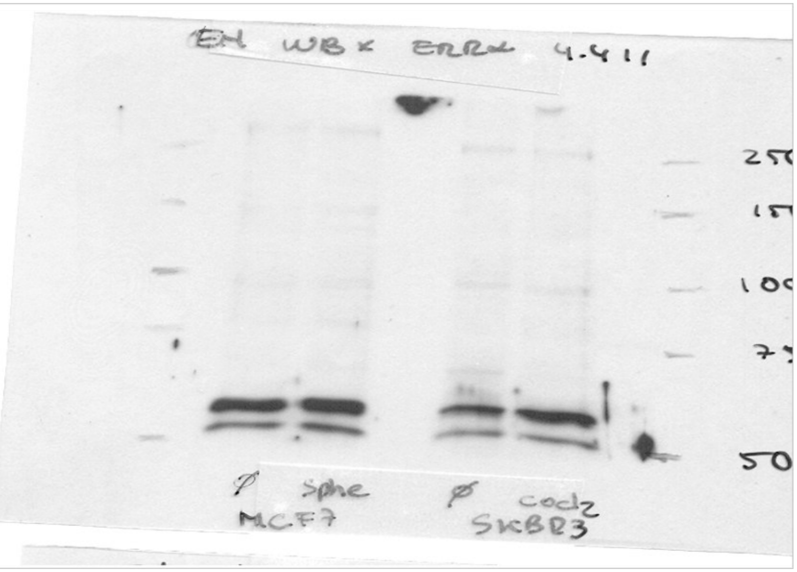

Full uncropped blot from **Fig. 1A**, ERRα.

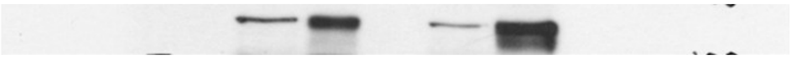

Original blot from **Fig. 1A**, PGC-1α. (The full image of this blot is not available.)

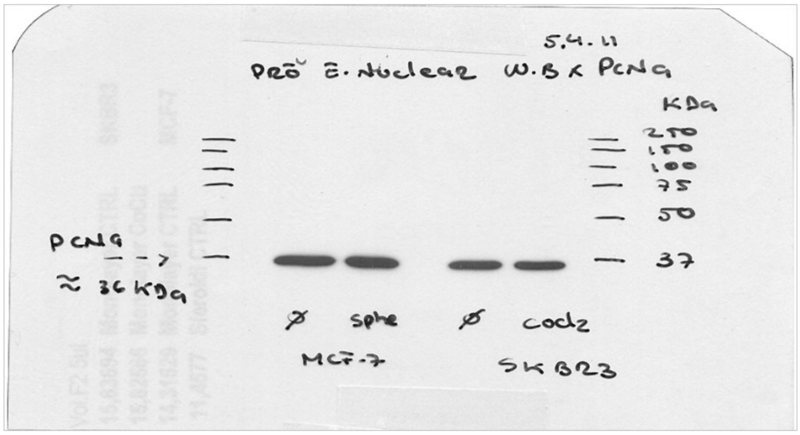

Full uncropped blot from **Fig. 1A**, PCNA.

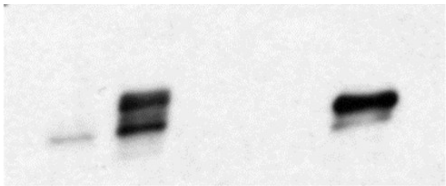

Original blot from **Fig. 1C**. (The full image of this blot is not available.)

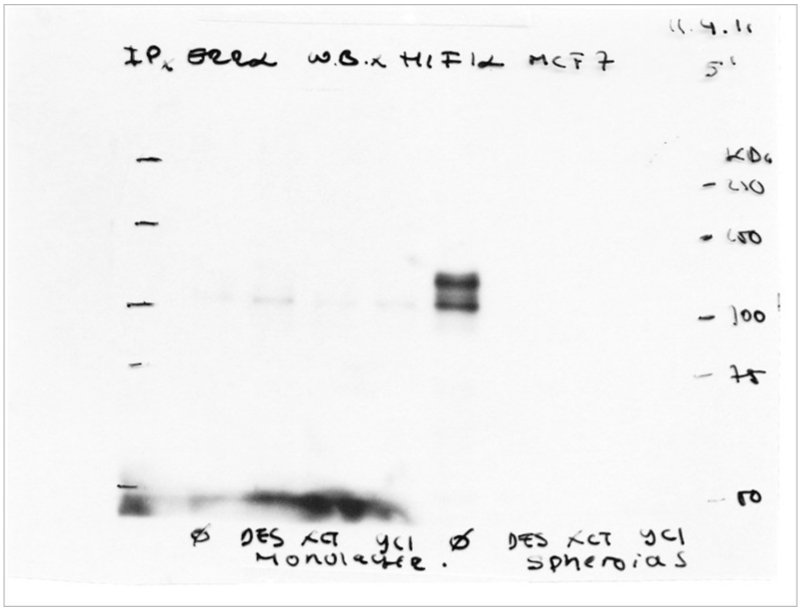

Full uncropped blot from **Fig. 1D**, MCF-7.

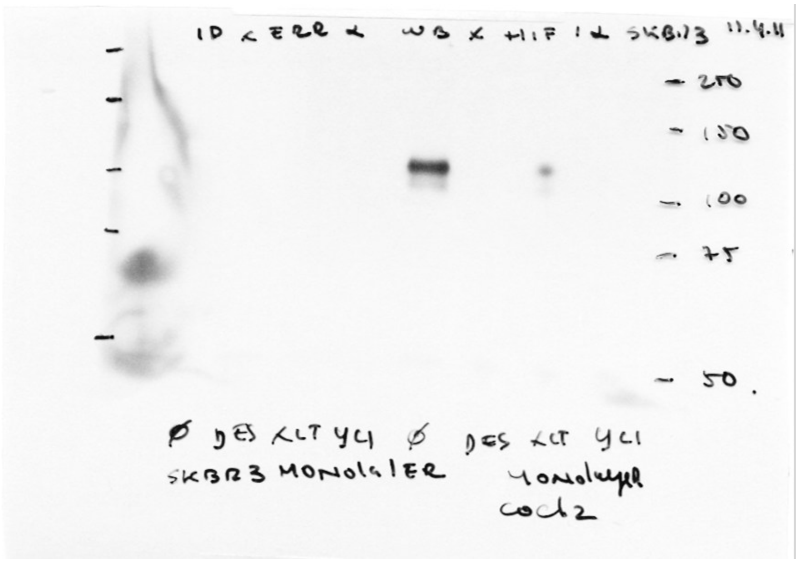

Full uncropped blot from **Fig. 1D**, SK-BR-3.

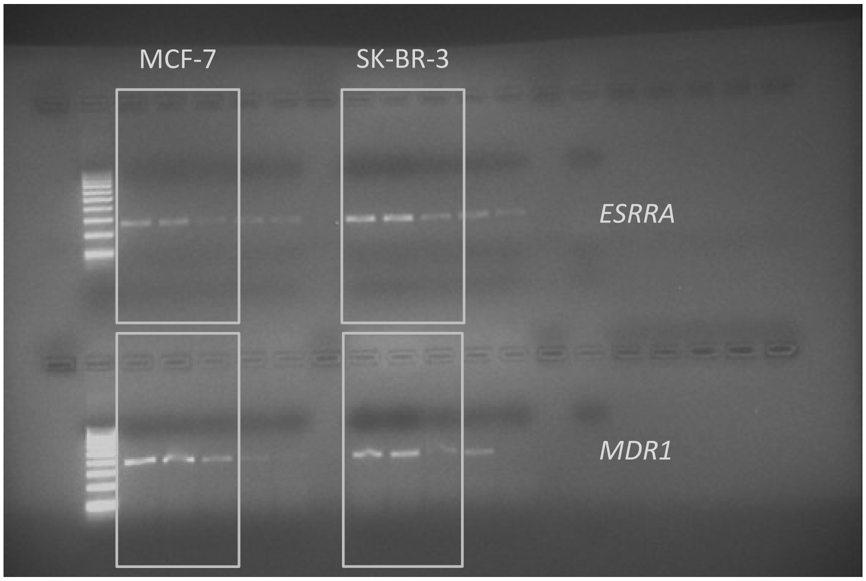

Full uncropped gel from **Fig. 3B**, *ESRRA* and *MDR1* in both cell lines.

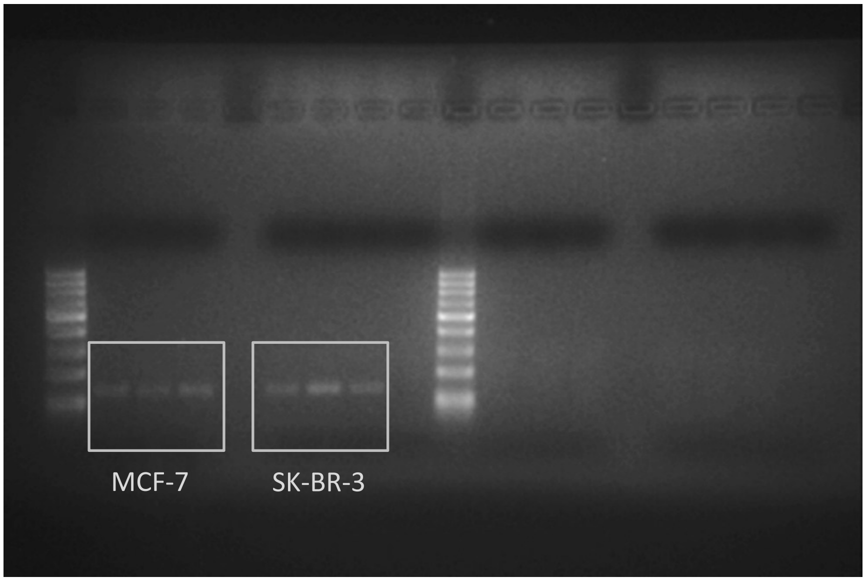

Full uncropped gel from **Fig. 3B**, *B2M* in both cell lines.

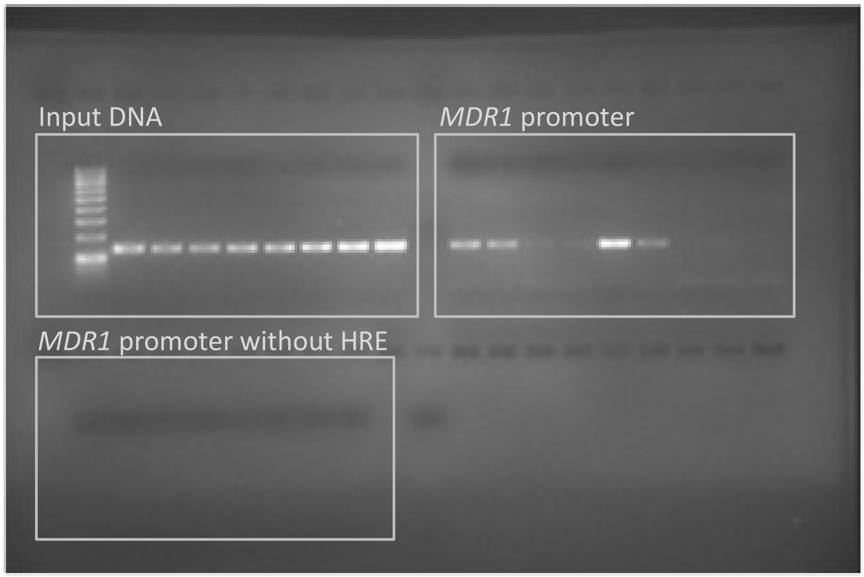

Full uncropped gel from **Fig. 4A**, Input DNA, *MDR1* promoter, *MDR1* promoter without HRE, MCF-7.

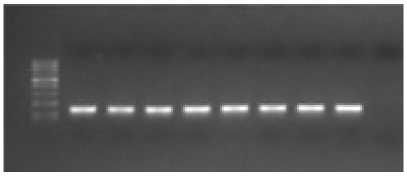

Full uncropped gel from **Fig. 4A**, *B2M*, MCF-7.

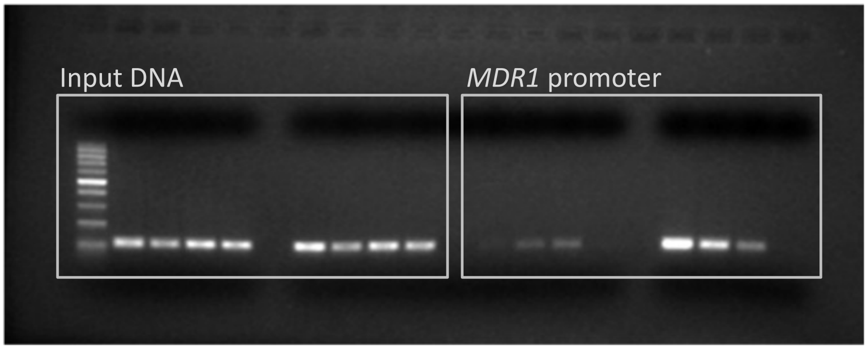

Full uncropped gel from **Fig. 4A**, Input DNA and *MDR1* promoter, SK-BR-3.

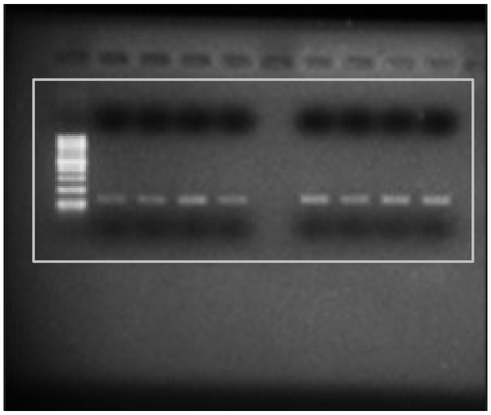

Full uncropped gel from **Fig. 4A**, *B2M*, SK-BR-3.

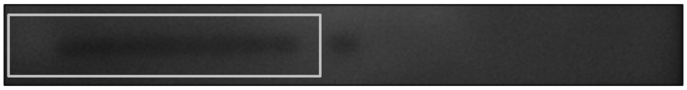

Original gel from **Fig. 4A**, *MDR1* promoter without HRE, SK-BR-3. (The full image of the gel is not available.)

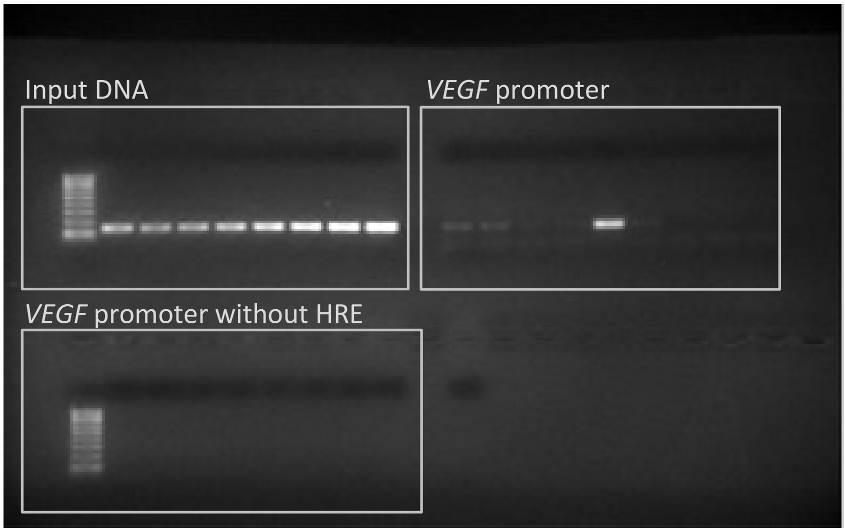

Full uncropped gel from **Fig. 4B**, Input DNA, *VEGF* promoter, *VEGF* promoter without HRE, MCF-7.

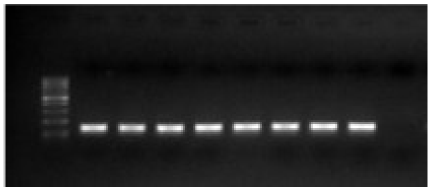

Full uncropped gel from **Fig. 4B**, *B2M*, MCF-7.

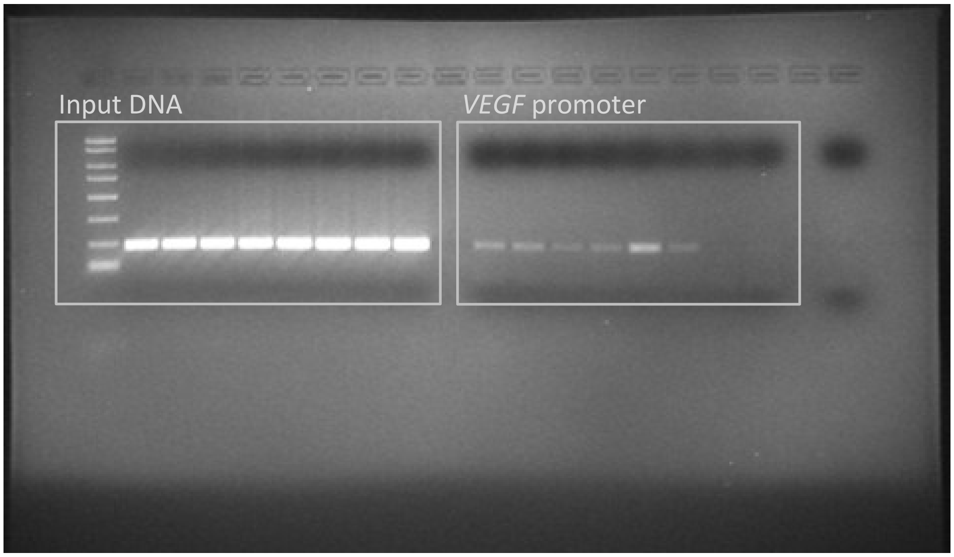

Full uncropped gel from **Fig. 4B**, Input DNA and *VEGF* promoter, SK-BR-3.

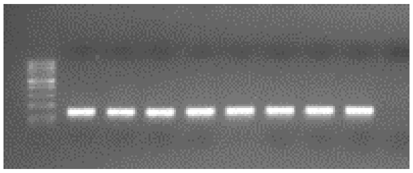

Full uncropped gel from **Fig. 4B**, *B2M*, SK-BR-3.

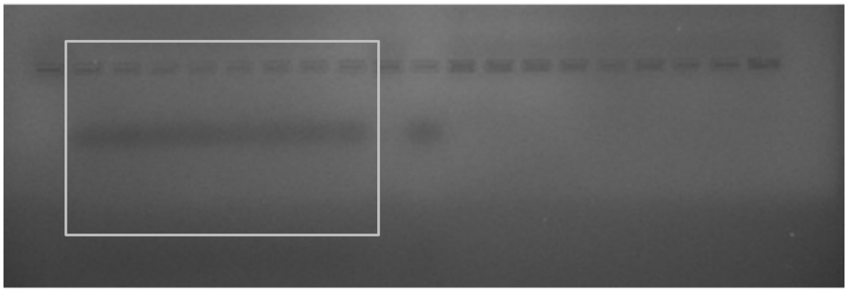

Full uncropped gel from **Fig. 4B**, *VEGF* promoter without HRE, SK-BR-3.
