# Supplementary material for: ERRα and HIF-1α Cooperate to Enhance Breast Cancer Aggressiveness and Chemoresistance Under Hypoxic Conditions
Source: Cancers (Basel). 2025 Jul 18;17(14):2382. doi: 10.3390/cancers17142382 (PMC12293820; doi:10.3390/cancers17142382)
Supplement: Supplementary file 1 [file cancers-17-02382-s001.zip › cancers-3728766-supplementary.pdf]

Supplementary Table S1. Description of TMA-included samples and IHC scores for ERRα, PGC-1, HIF-1α, and P-gP.

ID	Pathology diagnosis	TNM	Grade	Stage	ER score	ER %	PR score	PR %	HER2 score	Ki67 %t	ERRα score	PGC-1 score	HIF1α score	P-gP score
A1	Invasive carcinoma of no special type	T2N0M0	1-2	IIA	1	50	0	0	3	67	2	2	2	2
A2	Invasive carcinoma of no special type	T2N0M0	1-2	IIA	3	60	3	60	2	0	0.5	0.5	0.5	1
A3	Invasive carcinoma of no special type	T1N0M0	1	IA	3	90	0	0	2	0	1	1	2	0.5
A4	Invasive carcinoma of no special type	T4N0M0	2	IIIB	2	100	2	80	3	0	1	1	1	1
A5	Invasive carcinoma of no special type	T2N0M0	1	IIA	3	100	2	80	2	0	N/A	N/A	N/A	N/A
A6	Invasive carcinoma of no special type	T2N0M0	1	IIA	3	80	2	80	1	3	1	2	0.5	0.5
A7	Invasive carcinoma of no special type with necrosis	T2N0M0	1	IIA	0	0	0	0	3	0	0.5	0.5	1	1
A8	Invasive carcinoma of no special type	T2N0M0	2	IIA	3	100	3	90	2	30	0.5	1	0.5	0.5
A9	Invasive carcinoma of no special type	T2N0M0	2	IIA	3	100	3	100	1	2	1	2	2	2
A10	Invasive carcinoma of no special type	T1N0M0	1	IA	0	0	0	0	3	5	N/A	N/A	N/A	N/A
A11	Invasive carcinoma of no special type	T2N0M0	2	IIA	3	100	3	100	1	2	1	1	1	1
A12	Invasive carcinoma of no special type (sparse)	T1N0M0	1	IA	3	100	3	90	3	0	2	1	2	1
A13	Invasive carcinoma of no special type	T2N0M0	2	IIA	0	0	0	0	2	10	2	2	0.5	1
A14	Invasive carcinoma of no special type	T2N0M0	2	IIA	3	100	0	0	2	20	N/A	3	1	2
A15	Invasive carcinoma of no special type	T2N1M0	2	IIB	0	0	0	0	3	2	N/A	4	2	2
A16	Invasive carcinoma of no special type	T2N0M0	2	IIA	0	0	1	2	2	0	1.5	3	1	1
B1	Invasive carcinoma of no special type	T2N1M0	1	IIB	3	100	3	80	1	3	2	2	1	1
B2	Invasive carcinoma of no special type	T2N0M0	1	IIA	0	0	0	N/A	0	2	1.5	3	2	2
B3	Invasive carcinoma of no special type	T2N0M0	1	IIA	3	80	3	65	3	0	1	1	0.5	0.5
B4	Invasive carcinoma of no special type	T3N0M0	1	IIB	0	0	0	0	1	0	0.5	0.5	0	0.5
B5	Invasive carcinoma of no special type	T1N0M0	1	IA	3	80	2	70	2	10	2.5	2	2	N/A
B6	Invasive carcinoma of no special type with necrosis	T2N0M0	2	IIA	0	0	0	0	3	2	1.5	2	1	1
B7	Invasive carcinoma of no special type	T2N0M0	2	IIA	0	0	0	0	3	0	2	1	1	1
B8	Invasive carcinoma of no special type	T1N0M0	2	IA	2	80	2	70	2	15	1	0.5	0.5	1
B9	Invasive carcinoma of no special type	T2N0M0	2	IIA	0	0	0	0	1	0	2	1	1	1
B10	Invasive carcinoma of no special type	T2N0M0	2	IIA	2	70	1	20	2	3	2	2	1	1
B11	Invasive carcinoma of no special type	T2N0M0	1	IIA	3	60	2	40	2	0	1.5	2	1	2
B12	Invasive carcinoma of no special type	T4N0M0	2	IIIB	3	100	3	100	1	10	N/A	N/A	N/A	2
B13	Invasive carcinoma of no special type	T2N0M0	2	IIA	2	90	1	2	2	20	1.5	2	1	1
B14	Invasive carcinoma of no special type	T2N1M0	1	IIB	3	100	2	15	1	0	3	2	1	0.5

B15	Invasive carcinoma of no special type	T4N0M0	1	IIIB	3	100	3	100	1	5	0.5	1	0.5	1
B16	Invasive carcinoma of no special type	T2N0M0	1	IIA	3	100	3	100	1	10	0.5	1	1	1
C1	Invasive carcinoma of no special type	T3N0M0	1	IIB	0	0	0	0	3	0	2.5	3	1	1
C2	Invasive carcinoma of no special type	T2N0M0	1	IIA	0	0	0	0	1	10	1.5	2	0.5	0.5
C3	Invasive carcinoma of no special type	T2N1M0	1	IIB	1	90	1	15	3	0	2	3	0.5	3
C4	Invasive carcinoma of no special type	T2N1M0	1	IIB	3	80	3	80	2	0	1.5	2	1	2
C5	Invasive carcinoma of no special type	T2N1M0	1	IIB	3	100	2	70	1	15	2.5	2	1	3
C6	Invasive carcinoma of no special type	T2N0M0	1	IIA	3	100	3	100	0	5	2.5	4	2	3
C7	Invasive carcinoma of no special type	T3N0M0	2	IIB	0	0	0	0	0	0	3	2	2	3
C8	Invasive carcinoma of no special type	T2N0M0	2	IIA	3	90	3	90	3	0	2	2	1	2
C9	Invasive carcinoma of no special type	T2N0M0	2	IIA	0	0	2	15	1	2	2.5	1	2	2
C10	Invasive carcinoma of no special type	T2N1M0	2	IIB	0	0	0	0	3	5	3	1	2	1
C11	Invasive carcinoma of no special type	T2N1M0	2	IIB	3	100	2	30	3	10	2	0.5	1	2
C12	Invasive carcinoma of no special type	T2N1M0	2	IIB	0	0	N/A	N/A	3	10	1.5	2	1	1
C13	Invasive carcinoma of no special type	T2N1M0	N/A	IIB	2	80	1	2	1	0	2.5	1	1	0.5
C14	Invasive carcinoma of no special type	T2N1M0	2	IIB	0	0	0	0	3	5	0.5	1	0.5	0.5
C15	Invasive carcinoma of no special type	T2N0M0	2	IIA	0	0	0	0	N/A	0	3.5	1	0.5	1
C16	Invasive carcinoma of no special type	T2N1M0	2	IIB	0	0	0	0	N/A	0	3.5	2	3	2
D1	Invasive carcinoma of no special type	T2N0M0	2	IIA	2.5	70	3	60	3	15	1.5	2	1	1
D2	Invasive carcinoma of no special type	T2N1M0	2	IIB	0	0	0	0	3	5	0.5	1	0.5	0.5
D3	Invasive carcinoma of no special type	T4N1M0	2	IIIB	2	60	3	60	3	30	1.5	3	1	1
D4	Invasive carcinoma of no special type	T4N2M0	2	IIIB	2	80	3	80	1	15	0.5	1	1	1
D5	Invasive carcinoma of no special type	T3N0M0	2	IIB	2	50	0	0	2	0	3	4	4	3
D6	Invasive carcinoma of no special type	T3N0M0	2	IIB	1	5	0	0	3	20	1	3	2	2
D7	Invasive carcinoma of no special type	T2N0M0	2	IIA	3	90	0	0	3	10	1	1	1	1
D8	Invasive carcinoma of no special type	T4N2M0	2	IIIB	2	80	3	60	2	5	1	0.5	0.5	1
D9	Invasive carcinoma of no special type	T2N1M0	2	IIB	2.5	80	0	0	3	20	1.5	2	1	2
D10	Invasive carcinoma of no special type	T4N1M0	2	IIIB	1.5	15	0	0	3	30	1.5	2	1	2
D11	Invasive carcinoma of no special type	T2N0M0	2	IIA	3	100	3	100	1	10	1	1	2	1
D12	Invasive carcinoma of no special type	T2N0M0	2	IIA	0	0	0	0	3	15	2	2	1	2
D13	Invasive carcinoma of no special type	T2N0M0	2	IIA	2	70	3	50	3	20	1.5	1	0.5	1
D14	Invasive carcinoma of no special type	T3N1M0	2	IIIA	0	0	0	0	3	30	2	2	2	2
D15	Invasive carcinoma of no special type	T4N1M0	2	IIIB	3	100	1	5	3	15	2	2	2	2
D16	Invasive carcinoma of no special type	T4N1M0	2	IIIB	2	60	0	0	3	20	2	3	2	2
E1	Invasive carcinoma of no special type	T4N1M0	2	IIIB	2	50	0	0	2	5	2.5	2	1	1

E2	Invasive carcinoma of no special type	T2N1M0	2	IIB	0	0	0	0	3	0	2.5	3	1	1
E3	Invasive carcinoma of no special type	T2N1M0	2	IIB	0	0	0	0	3	10	2	3	2	2
E4	Invasive carcinoma of no special type (sparse)	T2N1M0	N/A	IIB	3	80	0	0	2	0	2	2	0.5	0.5
E5	Invasive carcinoma of no special type	T2N1M0	2	IIB	2	80	1	3	3	3	2	3	1	3
E6	Invasive carcinoma of no special type	T2N1M0	2	IIB	1	10	3	90	3	15	2.5	2	1	2
E7	Invasive carcinoma of no special type	T3N0M0	2	IIB	0	0	0	0	3	20	1.5	1	1	1
E8	Invasive carcinoma of no special type	T2N1M0	2	IIB	0	0	0	0	1	20	2	1	1	2
E9	Invasive carcinoma of no special type	T2N1M0	2	IIB	3	100	0	0	2	30	1.5	1	1	1
E10	Invasive carcinoma of no special type	T4N1M0	2	IIIB	1	5	0	0	2	0	2.5	2	2	2
E11	Invasive carcinoma of no special type	T2N0M0	2	IIA	3	90	3	80	3	5	1.5	0.5	1	1
E12	Invasive carcinoma of no special type	T3N0M0	2	IIB	0	0	1	5	3	20	1	1	2	2
E13	Invasive carcinoma of no special type	T2N0M0	2	IIA	1	20	0	0	3	20	2	2	2	2
E14	Invasive carcinoma of no special type	T4N1M0	N/A	IIIB	0	0	0	0	3	40	1.5	1	2	1
E15	Invasive carcinoma of no special type	T2N0M0	2	IIA	1	30	2	40	2	0	2	2	1	1
E16	Invasive carcinoma of no special type	T3N1M0	2	IIIA	2.5	80	0	0	3	15	1	2	2	1
F1	Invasive carcinoma of no special type	T2N1M0	2	IIB	N/A	N/A	N/A	N/A	N/A	N/A	2.5	N/A	N/A	N/A
F2	Invasive carcinoma of no special type	T3N0M0	2	IIB	1	10	0	0	3	5	2	2	2	2
F3	Invasive carcinoma of no special type	T2N1M0	2	IIB	3	100	3	100	2	15	2.5	2	1	0.5
F4	Invasive carcinoma of no special type	T3N0M0	2	IIB	3	100	1	5	1	10	2	2	1	2.5
F5	Invasive carcinoma of no special type	T3N1M0	2	IIIA	0	0	0	0	3	15	1.5	2	0.5	2.5
F6	Invasive carcinoma of no special type	T2N0M0	2	IIA	2	70	3	50	2	10	1.5	2	1	2
F7	Invasive carcinoma of no special type	T3N1M0	2	IIIA	3	90	1	5	2	15	1.5	3	2	2
F8	Invasive carcinoma of no special type	T2N0M0	2	IIA	3	10	0	0	3	20	1	1	2	1
F9	Invasive carcinoma of no special type	T2N0M0	2	IIA	3	100	3	100	3	15	0.5	0.5	1	1
F10	Invasive carcinoma of no special type	T2N0M0	2	IIA	1	10	1	10	2	10	2	2	3	1
F11	Invasive carcinoma of no special type	T3N1M0	2	IIIA	1	15	0	0	2	0	3.5	3	3	2
F12	Invasive carcinoma of no special type	T3N0M0	2	IIB	N/A	N/A	N/A	N/A	N/A	N/A	1.5	1	2	1
F13	Invasive carcinoma of no special type	T2N1M0	2	IIB	2.5	60	0	0	2	5	2.5	2	4	2
F14	Invasive carcinoma of no special type	T2N1M0	2	IIB	3	80	0	0	1	20	2.5	1	0.5	2
F15	Invasive carcinoma of no special type (sparse)	T2N1M0	2	IIB	0	0	0	0	1	25	2	2	2	1
F16	Invasive carcinoma of no special type with necrosis	T3N0M0	2	IIB	0	0	0	0	3	5	1.5	4	3	2
G1	Invasive carcinoma of no special type	T2N1M0	2	IIB	0	0	0	0	1	60	1.5	3	2	1
G2	Invasive carcinoma of no special type	T2N1M0	2	IIB	3	100	3	90	1	10	1.5	2	0.5	1
G3	Invasive carcinoma of no special type	T2N1M0	2	IIB	1	1	0	0	2	5	1.5	3	2	2

G4	Invasive carcinoma of no special type	T2N1M0	2	IIB	3	80	0	0	2	5	N/A	N/A	N/A	N/A
G5	Invasive carcinoma of no special type (sparse)	T3N1M0	N/A	IIIA	1	15	2	80	0	0	1.5	1	1	2
G6	Invasive carcinoma of no special type	T3N0M0	2	IIB	1	60	0	0	0	50	N/A	N/A	N/A	N/A
G7	Invasive carcinoma of no special type	T3N0M0	2	IIB	3	75	3	100	2	5	2	4	2	3
G8	Invasive carcinoma of no special type	T2N1M0	2	IIB	2.5	50	2.5	70	3	15	1.5	2	3	3
G9	Invasive carcinoma of no special type	T2N0M0	2	IIA	3	80	3	80	2	20	1	3	3	3
G10	Invasive carcinoma of no special type	T2N1M0	2	IIB	0	0	0	0	3	20	1	1	3	2
G11	Invasive carcinoma of no special type	T2N0M0	2	IIA	0	0	0	0	3	0	2	2	2	3
G12	Invasive carcinoma of no special type	T3N2M0	2	IIIA	0	0	0	0	1	15	1.5	2	2	2
G13	Invasive carcinoma of no special type	T2N1M0	2	IIB	0	0	0	0	1	5	2.5	2	2	1
G14	Invasive carcinoma of no special type	T2N0M0	2	IIA	0	0	0	0	2	50	1.5	3	1	3
G15	Invasive carcinoma of no special type	T2N0M0	2	IIA	0	0	0	0	2	60	2	1	1	1
G16	Invasive carcinoma of no special type	T2N0M0	2	IIA	0	0	0	0	1	65	2.5	3	2	2
H1	Invasive carcinoma of no special type	T3N0M0	3	IIB	0	0	0	0	0	15	4	3	2	1
H2	Invasive carcinoma of no special type	T3N0M0	2	IIB	0	0	0	0	2	10	0.5	1	1	0.5
H3	Invasive carcinoma of no special type	T2N1M0	2	IIB	0	0	0	0	2	20	1.5	2	0.5	2
H4	Invasive carcinoma of no special type	T1N0M0	2	IA	0	0	0	0	3	0	2.5	3	4	3
H5	Invasive carcinoma of no special type	T1N0M0	2	IA	0	0	0	0	1	20	1	2	0.5	1
H6	Invasive carcinoma of no special type	T2N0M0	2	IIA	0	0	0	0	N/A	15	1.5	2	1	2
H7	Invasive carcinoma of no special type	T4N1M0	2	IIIB	0	0	0	0	2	0	1	1	1	1
H8	Invasive carcinoma of no special type	T4N1M0	2	IIIB	1	10	0	0	0	0	1.5	2	2	2
H9	Invasive carcinoma of no special type	T2N0M0	3	IIA	0	0	0	0	1	30	1	1	2	2
H10	Invasive carcinoma of no special type	T2N0M0	3	IIA	0	0	0	0	2	80	1	0.5	2	0.5
H11	Invasive carcinoma of no special type	T2N0M0	3	IIA	0	0	0	0	3	20	0.5	1	1	1
H12	Invasive carcinoma of no special type	T2N0M0	2	IIA	0	0	0	0	3	10	1.5	2	2	1
H13	Invasive carcinoma of no special type	T2N0M0	2	IIA	1	20	1	2	N/A	0	1	1	2	1
H14	Invasive carcinoma of no special type	T3N0M0	2	IIB	0	0	0	0	1	0	3	2	2	1.5
H15	Invasive carcinoma of no special type	T4N0M0	2	IIIB	2	70	1	30	2	15	2	2	3	2
H16	Invasive carcinoma of no special type	T4N0M0	2	IIIB	1	55	3	60	1	0	2	2	2	2
I1	Invasive carcinoma of no special type	T2N0M0	3	IIA	0	0	0	0	2	10	1	1	0.5	0.5
I2	Invasive carcinoma of no special type	T4N0M0	2	IIIB	1	5	2	70	2	0	1	2	1	1
I3	Invasive carcinoma of no special type	T2N0M0	3	IIA	3	40	0	0	2	10	1.5	1	1	1
I4	Invasive carcinoma of no special type	T2N0M0	2	IIA	0	0	0	0	1	20	1	3	1	3
I5	Invasive carcinoma of no special type	T2N0M0	2	IIA	1	10	1	10	2	0	2	3	2	2
I6	Invasive carcinoma of no special type	T2N0M0	3	IIA	3	90	0	0	2	40	2	3	0.5	1

I7	Invasive carcinoma of no special type	T4N0M0	2	IIIB	3	100	3	80	1	20	1.5	3	1	2
I8	Invasive carcinoma of no special type	T2N1M0	3	IIB	0	0	0	0	3	20	2.5	2	1	1
I9	Invasive carcinoma of no special type	T1N0M0	2	IA	2	90	0	0	3	30	0.5	1	1	1
I10	Invasive carcinoma of no special type	T2N0M0	2	IIA	3	75	2	70	3	50	1.5	1	1	1
I11	Invasive carcinoma of no special type	T2N1M0	3	IIB	0	0	0	0	1	40	1.5	3	2	2
I12	Invasive carcinoma of no special type	T2N0M0	2	IIA	3	100	2	40	1	15	1	1	1	1
I13	Invasive carcinoma of no special type	T3N0M0	2	IIB	0	0	0	0	1	5	2.5	3	3	3
I14	Invasive carcinoma of no special type	T2N0M0	2	IIA	2	30	3	70	2	15	2	1	1	1
I15	Invasive carcinoma of no special type	T1N0M0	2	IA	0	0	0	0	3	20	2.5	4	3	2
I16	Invasive carcinoma of no special type	T2N1M0	2	IIB	3	100	2	50	3	30	3	4	3	4
J1	Invasive lobular carcinoma	T3N0M0	N/A	IIB	0	0	0	0	2	15	2	3	1	1
J2	Invasive lobular carcinoma	T2N1M0	N/A	IIB	1	30	1	2	2	0	3	2	1	1
J3	Invasive lobular carcinoma	T2N0M0	N/A	IIA	3	100	2	50	0	10	1.5	1	1	0.5
J4	Invasive lobular carcinoma	T2N0M0	N/A	IIA	3	100	3	90	3	15	2	3	2	3
J5	Invasive lobular carcinoma	T1N0M0	N/A	IA	3	100	2	60	2	0	1	1	1	1
J6	Invasive lobular carcinoma	T2N0M0	N/A	IIA	3	70	0	0	1	0	1	1	1	1
J7	Invasive lobular carcinoma	T2N0M0	N/A	IIA	3	80	0	0	1	0	1.5	1.5	2	1
J8	Invasive lobular carcinoma	T2N0M0	N/A	IIA	3	80	3	80	2	5	2	1.5	1	1
J9	Invasive lobular carcinoma	T4N0M0	N/A	IIIB	1	60	3	40	1	15	2	1	0.5	1.5
J10	Invasive lobular carcinoma	T2N0M0	N/A	IIA	2	50	0	0	1	0	2.5	1	2	0.5
J11	Invasive lobular carcinoma	T2N0M0	N/A	IIA	3	100	3	100	2	30	1.5	1	1	1
J12	Invasive lobular carcinoma	T2N0M0	N/A	IIA	3	65	3	50	1	0	2	2	2	2
J13	Invasive lobular carcinoma	T2N0M0	N/A	IIA	2	70	2	10	2	10	1.5	2	2	3
J14	Invasive lobular carcinoma	T3N0M0	N/A	IIB	3	100	3	100	0	40	1.5	2	1	2
J15	Invasive lobular carcinoma	T2N1M0	N/A	IIB	0	0	0	0	0	0	3	3	3	3
J16	Invasive lobular carcinoma (sparse)	T2N0M0	N/A	IIA	0	0	0	0	1	20	2.5	2	3	2
K1	Invasive lobular carcinoma	T2N1M0	N/A	IIB	2	40	0	0	3	0	2	3	3	1
K2	Invasive lobular carcinoma	T2N1M0	N/A	IIB	3	90	2	60	3	5	0.5	1	0.5	0.5
K3	Invasive lobular carcinoma	T2N0M0	N/A	IIA	2	90	0	0	2	0	2	1	0.5	0.5
K4	Invasive lobular carcinoma with necrosis	T2N1M0	N/A	IIB	3	80	0	0	0	0	3	2	1	2
K5	Invasive lobular carcinoma	T2N0M0	N/A	IIA	3	80	0	0	2	0	1.5	4	1	3
K6	Invasive lobular carcinoma	T4N1M0	N/A	IIIB	2	65	2	40	2	10	2.5	2	3	2
K7	Invasive lobular carcinoma	T2N1M0	N/A	IIB	2	50	2	30	1	0	1.5	1	1	1
K8	Invasive lobular carcinoma	T1N0M0	N/A	IA	3	90	3	80	1	20	1	1	0.5	1
K9	Breast tissue	N/A	N/A	N/A	0	0	1	N/A	0	0	2.5	2	2	1

K10	Breast tissue	N/A	N/A	N/A	N/A	N/A	N/A	N/A	N/A	N/A	3	1	1	0.5
K11	Adjacent normal breast tissue	N/A	N/A	N/A	1	N/A	1	N/A	0	0	3	3	3	3
K12	Adjacent normal breast tissue	N/A	N/A	N/A	N/A	N/A	1	N/A	0	0	2.5	2	2	3
K13	Adjacent normal breast tissue	N/A	N/A	N/A	1	N/A	1	N/A	0	0	2.5	2	3	2
K14	Adjacent normal breast tissue	N/A	N/A	N/A	1	N/A	1	N/A	0	0	N/A	N/A	N/A	3
K15	Adjacent normal breast tissue	N/A	N/A	N/A	1	N/A	1	N/A	0	0	N/A	N/A	N/A	2
K16	Adjacent normal breast tissue	N/A	N/A	N/A	1	N/A	1	N/A	0	0		2	N/A	2
L1	Adjacent normal breast tissue	N/A	N/A	N/A	N/A	N/A	N/A	N/A	N/A	N/A	1.5	2	0.5	0.5
L2	Adjacent normal breast tissue	N/A	N/A	N/A	N/A	N/A	N/A	N/A	N/A	N/A	N/A	N/A	0.5	0.5
L3	Adjacent normal breast tissue	N/A	N/A	N/A	1	N/A	0	0	0	0	1	1	1	1
L4	Adjacent normal breast tissue	N/A	N/A	N/A	1	N/A	0	0	0	0	1.5	1	1	1
L5	Adjacent normal breast tissue	N/A	N/A	N/A	1	N/A	1	N/A	0	0	2	0.5	0	0.5
L6	Adjacent normal breast tissue	N/A	N/A	N/A	1	N/A	1	N/A	0	0	1.5	0.5	0	0.5
L7	Adjacent normal breast tissue	N/A	N/A	N/A	1	N/A	1	N/A	0	0	2.5	2	0.5	1
L8	Adjacent normal breast tissue	N/A	N/A	N/A	N/A	N/A	N/A	N/A	N/A	0	2.5	1	0	0.5
L9	Adjacent normal breast tissue	N/A	N/A	N/A	1	N/A	1	N/A	0	0	2.5	1	1	2
L10	Adjacent normal breast tissue	N/A	N/A	N/A	1	N/A	2	N/A	0	0	2	N/A	N/A	3
L11	Adjacent normal breast tissue	N/A	N/A	N/A	1	N/A	1	N/A	0	0	1	1	0.5	1
L12	Adjacent normal breast tissue	N/A	N/A	N/A	1	N/A	2	N/A	0	0	1.5	2	1	1
L13	Adjacent normal breast tissue	N/A	N/A	N/A	1	N/A	2	N/A	0	0	2	0.5	0.5	1
L14	Adjacent normal breast tissue	N/A	N/A	N/A	1	N/A	2	N/A	0	0	2.5	2	1	2
L15	Adjacent normal breast tissue	N/A	N/A	N/A	1	N/A	1	N/A	0	0	2.5	2	2	3
L16	Adjacent normal breast tissue	N/A	N/A	N/A	1	N/A	1	N/A	0	0	2.5	3	N/A	3
M1	Adjacent normal breast tissue	N/A	N/A	N/A	N/A	N/A	N/A	N/A	N/A	N/A	2	1	0.5	0.5
M2	Adjacent normal breast tissue	N/A	N/A	N/A	2	N/A	1	N/A	0	0	2	0.5	0	0.5
M3	Adjacent normal breast tissue	N/A	N/A	N/A	2	N/A	2	N/A	0	0	0.5	0.5	0	0.5
M4	Adjacent normal breast tissue	N/A	N/A	N/A	2	N/A	2	N/A	0	0	1	1	0.5	1
M5	Adjacent normal breast tissue	N/A	N/A	N/A	2	N/A	2	N/A	0	0	3	1	1	2
M6	Adjacent normal breast tissue	N/A	N/A	N/A	1	N/A	1	N/A	0	0	3	N/A	2	N/A
M7	Adjacent normal breast tissue	N/A	N/A	N/A	N/A	N/A	N/A	N/A	N/A	N/A	1.5	0.5	0	1
M8	Adjacent normal breast tissue	N/A	N/A	N/A	1	N/A	1	N/A	0	0	1.5	1	1	1
M9	Adjacent normal breast tissue	N/A	N/A	N/A	1	N/A	1	N/A	0	0	2	1	0.5	1
M10	Adjacent normal breast tissue	N/A	N/A	N/A	1	N/A	1	N/A	0	0	2	1	0.5	2
M11	Adjacent normal breast tissue	N/A	N/A	N/A	1	N/A	1	N/A	0	0	2	2	2	2
M12	Adjacent normal breast tissue	N/A	N/A	N/A	1	N/A	1	N/A	0	0	2	1	1	2

M13	Adjacent normal breast tissue	N/A	N/A	N/A	1	N/A	1	N/A	0	0	2.5	2	1	2
M14	Adjacent normal breast tissue	N/A	N/A	N/A	1	N/A	1	N/A	0	0	2.5	N/A	2	N/A
M15	Adjacent normal breast tissue	N/A	N/A	N/A	N/A	N/A	N/A	N/A	N/A	N/A	0	1	0	1
M16	Adjacent normal breast tissue	N/A	N/A	N/A	N/A	N/A	N/A	N/A	N/A	N/A	1.5	2	N/A	2

N/A, not available because the core is invalid, or the value is either not applicable or negative in IHC markers.
